# FOXP3 Stability-Adaptation Paradox: Reshaping Treg-Based Therapies for Intestinal Diseases

**DOI:** 10.7150/ijbs.137066

**Published:** 2026-08-21

**Authors:** Yushuo Ren, Xiaohui Liu, Boya Wang, Haoyuan Yin, Jiatong Zhao, Shuzi Xin, Hongli Wang, Yuchen Zhang, Xinyue Liu, Jingyu Liu, Rongxuan Hua, Xiang Tian, Yuhang Bian, Sijin Gu, Haoxuan Zhang, Tong Wei, Lei Gao, Xin Lu, Hongwei Shang, Han Gao, Jingdong Xu

**Affiliations:** 1 Department of Clinical Medicine, School of Basic Medical Sciences, Capital Medical University, Beijing 100069, China.; 2 Department of Physiology and Pathophysiology, School of Basic Medical Sciences, Capital Medical University, Beijing 100069, China.; 3 Key laboratory of Carcinogenesis and Translational Research (Ministry of Education/Beijing), Department of Gastrointestinal Oncology, Peking University Cancer Hospital & Institute, Beijing 100142, China.; 4 Liver Research Centre, Beijing Friendship Hospital, Capital Medical University, Beijing Key Laboratory of Translational Medicine on Liver Cirrhosis, National Clinical Research Centre of Digestive Diseases, Beijing 100050, China; 5 School of Intelligent Medical Engineering, School of Biomedical Engineering, Capital Medical University, Beijing 100069, China.; 6 Experimental Centre for Morphological Research Platform, Capital Medical University, Beijing 100069, China.; 7 Department of Clinical Laboratory, Aerospace Centre Hospital, 100049 Beijing, China.

**Keywords:** Regulatory T cells, FOXP3, Inflammatory bowel disease, Colorectal cancer, Immunotherapy, FOXP3 stability, CAR-Tregs, microbiota

## Abstract

Regulatory T cells (Tregs) maintain intestinal immune homeostasis, but their therapeutic potential is constrained by a fundamental paradox: the same plasticity that enables tissue repair renders FOXP3 vulnerable to degradation in chronic inflammation. Mechanistically, microbial metabolites (short-chain fatty acids, bile acids) and retinoic acid stabilize FOXP3 and induce RORγt⁺/GATA3⁺ Treg specialization. In contrast, inflammatory cytokines and succinate accumulation drive ER stress and post-translational FOXP3 degradation, leading to lineage instability in inflammatory bowel disease, colorectal cancer, and celiac disease. Current Tregs-based therapies—adoptive transfer, low-dose IL-2, CAR-Tregs, and microbiota consortia—have demonstrated safety profiles yet exhibit limited efficacy due to this inherent instability. Next-generation strategies therefore focus on actively stabilizing FOXP3 (e.g., gut-restricted HDAC inhibitors) and engineering exhaustion-resistant CAR-Tregs. Three questions remain for clinical translation: how to preserve Treg stability without compromising anti-tumor immunity; which biomarkers (succinate, TSDR methylation, FOXP3Δ2/FL ratio) predict response; and whether logic-gated CAR-Tregs can overcome exhaustion. Addressing these challenges will enable the development of precision Treg immunotherapy for intestinal diseases.

## 1. Background

The gut immune system faces a unique challenge. It must remain tolerant to commensal microbes and dietary antigens while defending against pathogens. Regulatory T cells (Tregs), defined by the transcription factor FOXP3, are central to this balance 1, 2. Beyond classical immunosuppression, Tregs exhibit tissue-specific adaptations—including RORγt⁺ and GATA3⁺ specialization—and directly promote mucosal repair via amphiregulin (AREG) secretion 1-3. However, the same plasticity that enables these beneficial functions becomes a liability in chronic inflammation, where Tregs can lose FOXP3 expression and convert into pathogenic exFOXP3 Th17 cells. This stability-adaptation paradox lies at the heart of Treg dysfunction in intestinal diseases.

This framework is conceptually distinct from the established notion of Treg plasticity. Treg plasticity refers to the capacity of Tregs to lose FOXP3 expression and acquire alternative effector fates, such as exFOXP3 Th17 cells, under inflammatory conditions 1, 4. In contrast, the stability-adaptation paradox addresses a more fundamental functional tension: the same environmental cues that enable tissue-specific Treg specialization—microbial metabolites, retinoic acid, and tissue damage signals—also render FOXP3 vulnerable to post-translational degradation when these signals become dysregulated in chronic inflammation. Thus, while plasticity describes a cell fate switch, the stability-adaptation paradox explains a molecular vulnerability inherent to Treg adaptation 4. Importantly, this paradox is not restricted to intestinal Tregs; rather, it reflects a general principle of tissue-resident Treg biology. Tregs in diverse non-lymphoid tissues—including visceral adipose tissue, skeletal muscle, and the colonic lamina propria—undergo similar tissue-specific adaptations that enable local function but also create vulnerabilities to lineage instability under inflammatory stress 3, 5, 6. Given that the intestinal milieu exerts a particularly powerful influence on Treg functionality, we have chosen intestinal diseases as a representative paradigm to illustrate this framework in the present review.

Treg dysfunction - whether due to numerical deficiency, impaired inhibitory capacity, or lineage instability - is considered to be associated with a variety of intestinal diseases 7. Despite advances in biologics and small molecules, many patients fail to achieve durable remission, highlighting an urgent need for therapies that simultaneously control inflammation, promote healing, and restore immune tolerance 8. Tregs-based cellular immunotherapies (adoptive transfer, CAR-Tregs, Tr1 cells) offer a promising avenue 9, but their clinical translation has been hindered by a critical insight: expanding Treg numbers is insufficient if those cells remain unstable in the inflammatory milieu.

This review provides a comprehensive overview of the biological characteristics and dysfunction of Tregs in major digestive disorders, alongside current therapeutic strategies targeting this cell population. We then discuss key challenges including instability, tumor immune escape, interpatient variability, and biomarker deficits, and propose a unifying framework centered on FOXP3 protein stabilization as the next frontier for precision immunotherapy of the gut.

## 2. Phenotypic characteristics of Tregs

### 2.1 Phenotypic identity and lineage heterogeneity

CD4^+^Tregs are defined by constitutive high expression of CD25 and the transcription factor FOXP3 10, 11. FOXP3 is a key regulatory factor for the development and function of CD4^+^CD25^+^ regulatory T cells 11-14. Additionally, Tregs constitutively highly expresses cytotoxic T lymphocyte-associated antigen 4 (CTLA-4) on its surface, a key mediator of immunosuppression.

Based on developmental origin, CD4⁺ Tregs are classified into thymus-derived Tregs (tTregs, also called nTregs) and peripherally induced Tregs (pTregs). tTregs arise from CD4⁺ thymocytes 15, pTregs develop from naïve CD4^+^ T cells in peripheral tissues under TGF-β and IL-2 stimulation 16-18. tTregs primarily enforce self-tolerance, whereas pTregs maintain tolerance to environmental antigens (commensal microbiota, diet, and tumor neoantigens). Tregs can also be induced *in vitro* (iTregs) from naïve CD4⁺ T cells upon TCR stimulation with TGF-β and IL-2. However, iTregs exhibit incomplete CpG demethylation at the *FOXP3* locus, leading to unstable FOXP3 expression and limiting their functional relevance 19, 20.

The molecular markers of nTregs and p/iTregs have been gradually clarified. Studies have shown that in nTregs, the transcription factor Helios and the cell surface molecule Neuropilin-1 are highly expressed selectively, while in p/iTregs, they are lowly expressed 21-23, and can serve as markers for thymus-derived Tregs.

Beyond FOXP3⁺ Tregs, type 1 regulatory T (Tr1) cells do not express FOXP3 but suppress via highly secreting IL-10 and TGF-β 24, 25. Recent studies have revealed that the transcription factor Eomesodermin can define a subset of Tr1-like cells with both cytotoxic and immunosuppressive functions 26, providing a new perspective for understanding the heterogeneity of Tr1. Table 1 summarizes the phenotypic and functional heterogeneity of major Treg subsets.

### 2.2 Developmental and stabilizing regulatory networks

The establishment and maintenance of the Treg lineage are governed by a multi-level regulatory network that spans thymus development and extends through transcriptional regulation, signal transduction, metabolic adaptation, and stress responses.

#### 2.2.1 Thymus development and lineage commitment

**Figure 1:**
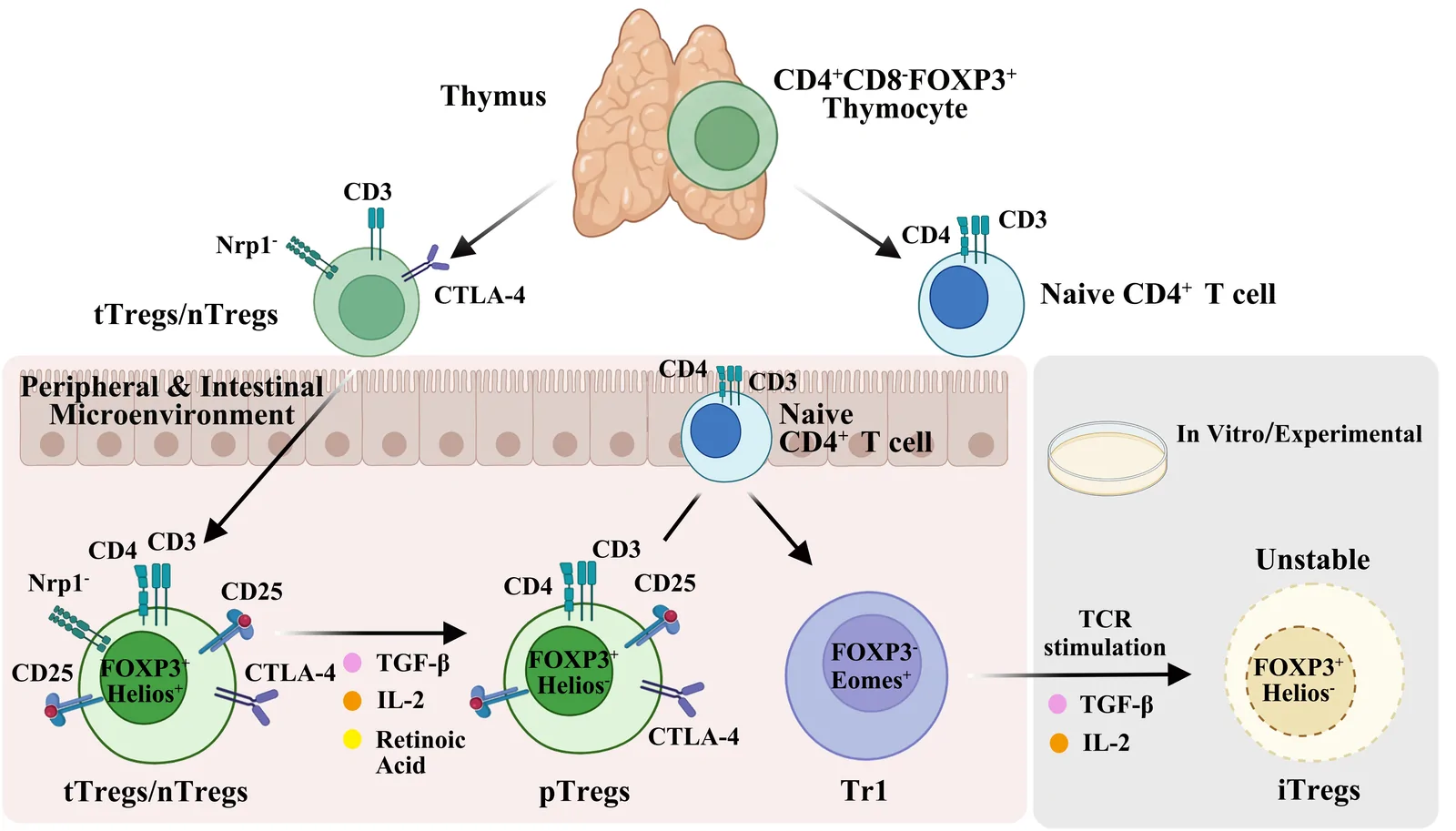
** Developmental origins and phenotypic characteristics of regulatory T cell subsets.** Thymus-derived Tregs (tTregs/nTregs) develop from CD4⁺CD8⁻FOXP3⁺ thymocytes and express robust Nrp1, CD3, and CTLA-4. In the peripheral and intestinal microenvironment, tTregs/nTregs (FOXP3⁺Helios⁺) can be induced by TGF-β, IL-2, and retinoic acid to differentiate into pTregs (FOXP3⁺Helios⁻). Naïve CD4⁺ T cells can also differentiate into Tr1 cells (FOXP3⁻Eomes⁺) in the periphery. Under in vitro conditions, naïve T cells stimulated with TCR in the presence of TGF-β and IL-2 become iTregs (FOXP3⁺Helios⁻Nrp1⁻), which exhibit phenotypic instability.

Treg lineage commitment begins in the thymus, where developing Tregs acquire stable FOXP3 expression through a multi-stage selection process. Helios, expressed from the double-negative 2 stage onward in all CD4⁺CD8⁻FOXP3⁺ thymocytes, serves as a stable lineage marker for thymus-derived Tregs (tTregs) 21. This early commitment establishes epigenetic stability and distinguishes tTregs from peripherally induced Tregs (pTregs), which arise from naive CD4⁺ T cells upon TGF-β and IL-2 stimulation 16-18 (Figure 1).

#### 2.2.2 Transcriptional control of FOXP3

Once the lineage is established, a multi-layered network of transcription factors, signaling pathways, and metabolic adaptations ensures Tregs identity and functional integrity.

FOXP3 expression is precisely controlled by multiple conserved non-coding sequences (CNSs). Different metabolites act selectively on these enhancers—a concept termed “enhancer selectivity.” Short-chain fatty acids promote Tregs differentiation via CNS1, whereas the bile acid metabolite isoalloLCA acts through CNS3 27.

Beyond CNS-dependent regulation, FOXP3 transcription is orchestrated by a broader network of transcription factors that operate upstream of, concurrently with, or downstream of FOXP3 itself 28. NFAT plays a pivotal role by forming cooperative complexes with FOXP3 to mediate Treg suppressive function—switching from AP-1 partnership that drives T cell activation to FOXP3 partnership that executes the suppressor program. BATF, an AP-1 family member, enhances FOXP3-chromatin binding in activated and tumor-infiltrating Tregs and is required for the development of non-lymphoid tissue Treg precursors. c-Rel acts as a pioneer factor for Treg lineage commitment by binding CNS regions of the FOXP3 locus. FOXO1 and FOXO3 integrate extrinsic signals to control Treg development; their deficiency impairs Treg generation, and both proteins directly bind the Foxp3 locus to regulate promoter activity. ETS1 positively regulates FOXP3 transcription and binds to the TSDR exclusively in its demethylated state 29. BACH2 enforces Treg lineage integrity by repressing effector programs and maintaining immune homeostasis 28.

Transcription factor interactions further refine Treg function. RORγt stabilizes FOXP3 by repressing T-bet, thereby preventing Th1-like conversion 30. c-MAF collaborates with RORγt to drive IL-10 production 31, while GATA3 inhibits RORγt-mediated IL-17 production, preserving Treg stability in inflammatory environments 32. These mutually reinforcing interactions constitute the molecular basis for Treg functional specialization.

IL-2 activates the phosphorylation of STAT5 (pSTAT5) by binding to its receptor, thereby regulating the survival and function of Treg 41. STAT5a/b bind directly to conserved STAT5-binding sites within the Foxp3 promoter in CD4+CD25+ Treg, but not in conventional T cells 33, 34. In contrast, STAT3 mediates IL-6-dependent FOXP3 downregulation, indicating opposing roles for STAT5 and STAT3 in Foxp3 regulation 33, 34. The Runx1-Cbfβ complex further reinforces this network by binding CNS regions of the Foxp3 gene and maintaining constitutive FOXP3 expression 35.

The IL-4/STAT6 axis negatively modulates Treg stability: STAT6 deficiency results in iTregs that maintain a stable phenotype with high Foxp3 and CD25 expression even under inflammatory conditions, associated with increased TSDR demethylation and reduced DNMT1 expression 36. Pharmacological STAT6 inhibition with AS1517499 enhances iTreg stability, maintaining high expression of Foxp3, CD25, PD-1, and CTLA-4 for up to 10 days, even under inflammatory conditions 37. These findings establish STAT6 as a critical negative regulator of Treg stability and a promising therapeutic target for enhancing Treg-based therapies.

Epigenetic modifications provide an additional layer of FOXP3 control 28. The Treg-specific demethylated region (TSDR)—a conserved CpG-rich element within the FOXP3 locus—is selectively demethylated during early thymic Treg development via TET enzymes, and this imprinting is essential for stable FOXP3 expression and long-term lineage stability. The methylation state of TSDR controls its transcriptional activity: demethylated TSDR acts as an enhancer, whereas methylated TSDR fails to support transcription. ETS1 binds to the TSDR only in its demethylated state, and disruption of its binding sites drastically reduces enhancer activity 29. At the cis-regulatory level, CNS0 and CNS3 are hierarchically activated prior to FOXP3 promoter activation via sequential genomic looping; their double deletion completely abrogates Treg generation and impairs FOXP3 stability 28. Furthermore, STAT6 signaling and TSDR methylation are functionally intertwined, as STAT6 deficiency combined with CRISPR-TET1-mediated TSDR demethylation enhances Foxp3 expression and iTreg suppressive capacity 36.

Histone modifications provide an additional layer of regulation. Foxp3 protein itself is subject to acetylation at lysine residues within its forkhead domain. HDAC inhibitor treatment increases Foxp3 acetylation, enhances Foxp3 binding to target gene promoters such as IL2, and suppresses endogenous IL-2 production; among class I and II HDACs, HDAC9 is particularly important, as its deficiency results in enhanced Foxp3 acetylation, increased Treg suppressive function, and reduced susceptibility to colitis 38. CNS2/TSDR serves as a critical cis-regulatory element that maintains Foxp3 expression during cell division by sensing IL-2/STAT5 signaling, enabling dividing Tregs to sustain Foxp3 under limiting IL-2 conditions and counteracting proinflammatory cytokine-driven silencing of the Foxp3 locus 39. The convergence of these epigenetic mechanisms—TSDR demethylation, histone acetylation, and CNS-mediated enhancer coordination—on FOXP3 stability underscores the importance of targeting the epigenome, rather than merely expanding Treg numbers, to achieve durable Treg stability for therapeutic applications.

This multilayered transcriptional and epigenetic network—encompassing NFAT/AP-1 switching, FOXO-mediated lineage commitment, BACH2-dependent integrity enforcement, ETS1-mediated TSDR enhancer activation, STAT5-driven transcriptional activation, Runx1-Cbfβ-mediated epigenetic locking, STAT6-driven TSDR methylation, and CNS0/CNS3-mediated enhancer coordination—collectively ensures FOXP3 expression fidelity. Importantly, the convergence of these pathways on FOXP3 stability provides the molecular rationale for why next-generation Treg-based therapies must move beyond mere numerical expansion toward active FOXP3 stabilization—a theme we develop in subsequent sections.

#### 2.2.3 Metabolic and stress adaptation

Signaling pathways further reinforce Tregs identity. IL-2 activates the phosphorylation of STAT5 (pSTAT5) by binding to its receptor, thereby regulating the survival and function of Tregs 40. TGF-β signaling is also necessary for pTregs induction and works synergistically with retinoic acid to promote the differentiation of naïve T cells into FOXP3^+^ Tregs 41, 42.

Metabolic adaptation provides an additional layer of refinement. Tissue-resident Tregs upregulate arginase 2 (ARG2), which inhibits mTOR signaling to enhance suppressive function and improve survival in inflammatory tissues 43. This not only improves the inhibitory ability of Tregs but also increases their adaptability and survival advantage in inflammatory tissues.

The stability of Tregs in an inflammatory environment is ensured by multiple mechanisms. Endoplasmic reticulum (ER) stress promotes the downregulation of FOXP3 by activating the IRE1α-p38 axis, while the E3 ligase Hrd1 counteracts this pathway. Treg-specific Hrd1 deficiency leads to spontaneous intestinal inflammation in mice 44. Similarly, TMED4 maintains the function of Tregs by stabilizing IRE1α and activating the NRF2-dependent antioxidant response, its absence results in unstable FOXP3 and severe colitis 45.

TMED4 serves as a universal stabilizer of Treg suppressive function through the IRE1α-ROS-NRF2 axis 45. Consequently, selective ablation of TMED4 in Tregs consistently impairs their stability and suppressive capacity, leading to effector T cell hyperactivation. Notably, the opposing outcomes observed in different disease contexts—enhanced antitumor immunity in cancer versus aggravated inflammation in IBD and EAE—are both attributable to the the same underlying mechanism, which is the loss of Treg-mediated immune suppression unleashes effector T cells. While this effect is therapeutically beneficial in malignancy it proves deleterious in autoimmune disorders. This context-dependent effects thus underscores the critical need to consider the pre-existing immunological milieu when interpreting Treg-targeted interventions.

At the metabolic level, elevated succinate in the inflammatory environment inhibits the production of succinyl-CoA mediated by DLST, reduces the succinyl modification of FOXP3, and promotes its ubiquitination and degradation 46. This succinate-OGDHc-FOXP3 axis (detailed in Section 5.1) directly links a disease-elevated metabolite to Tregs functional collapse.

Together, this multilayered network—normally protective—becomes the very substrate for FOXP3 instability when inflammatory signals overwhelm it. The convergence of protective and disruptive signals on FOXP3 protein stability lies at the heart of the stability-adaptation paradox (Box 1).

### 2.3 Translational Considerations: Murine vs. Human Treg Biology

While mouse models have provided foundational insights into Treg development, stability, and function, significant interspecies differences must be considered when translating experimental findings to human intestinal diseases.

In mice, Helios and Neuropilin-1 (Nrp1) are widely used to distinguish tTregs from pTregs 21-23, 47. However, in humans, neither marker is definitive: Helios is expressed in a substantial proportion of CD8⁺ T cells 48, and Nrp1 expression on human Tregs is more variable and context-dependent 48. Moreover, CD49f (integrin α6) has recently been identified as a marker defining functionally heterogeneous human Treg subsets—CD49f⁺ Tregs display reduced suppressive markers (decreased Helios and granzyme B) yet enhanced IL-10 production, and this functional heterogeneity is lost in autoimmune diseases such as SLE 49, indicating that human Treg markers reflect functional diversity not captured by mouse models.

Beyond phenotypic markers, IL-2-driven Treg heterogeneity exhibits notable species-specific differences. In mice, IL-2 signaling is essential for Treg maintenance, but human IL-2-responsive Treg subsets exhibit more complex heterogeneity. Raeber et al demonstrated that in SLE patients receiving low-dose IL-2 immunotherapy, CD38⁺, HLA-DR⁺, and double-positive Treg subsets are preferentially expanded, each with distinct tissue-homing properties: CD38⁺ Tregs express gut-homing receptors (integrin α4β7, CCR9), HLA-DR⁺ Tregs express skin-homing receptors (CLA, CCR4), and double-positive Tregs express CXCR3 for homing to inflammatory sites. This tripartite subset specialization—with distinct activation programs, transcriptional regulators (BATF, IRF4), and clonal relationships—has not been described in mouse IL-2 responses, highlighting species-specific differences in Treg functional diversification 50.

Functional heterogeneity and tissue adaptation further distinguish human Tregs from their murine counterparts. Human Treg clones with identical antigen specificity can exhibit distinct functional clusters (e.g., cytolytic, cytokine-producing, or infectious tolerance-inducing), a level of heterogeneity often overlooked in bulk-assayed murine studies 51. Human CD49f⁺ Tregs possess unique functional characteristics—reduced classical suppressive markers but enhanced IL-10 production—and are disrupted in SLE, suggesting that human Treg functional heterogeneity is both more nuanced and more disease-relevant 49. Additionally, the composition of human small intestinal intraepithelial lymphocytes includes unique CD8⁺ Treg populations that are reduced in active celiac disease 52, indicating tissue-specific adaptation patterns not fully recapitulated in mice.

Regarding plasticity and stability, while mouse models have elucidated core ER stress and succinate pathways, human Tregs exhibit additional complexity: transient FOXP3 expression in activated non-regulatory T cells, alternative splicing (FOXP3Δ2/FL ratio), and the necessity of functional validation, as no single marker can exclusively define all human Tregs 51. The IL-2-driven distinct activation programs (CD38⁺, HLA-DR⁺, double-positive) further illustrate that human Treg stability and function are governed by multi-layered, context-dependent mechanisms that may not directly mirror murine systems 51.

In summary, while mouse models provide invaluable mechanistic insights, their translation to human therapies requires careful validation of the specific human Treg subsets and pathways involved, ideally integrating transcriptomic, epigenetic, and functional readouts while accounting for source- and tissue-specific heterogeneity.

## 3. Functional modalities and suppressive mechanisms

**Figure 2:**
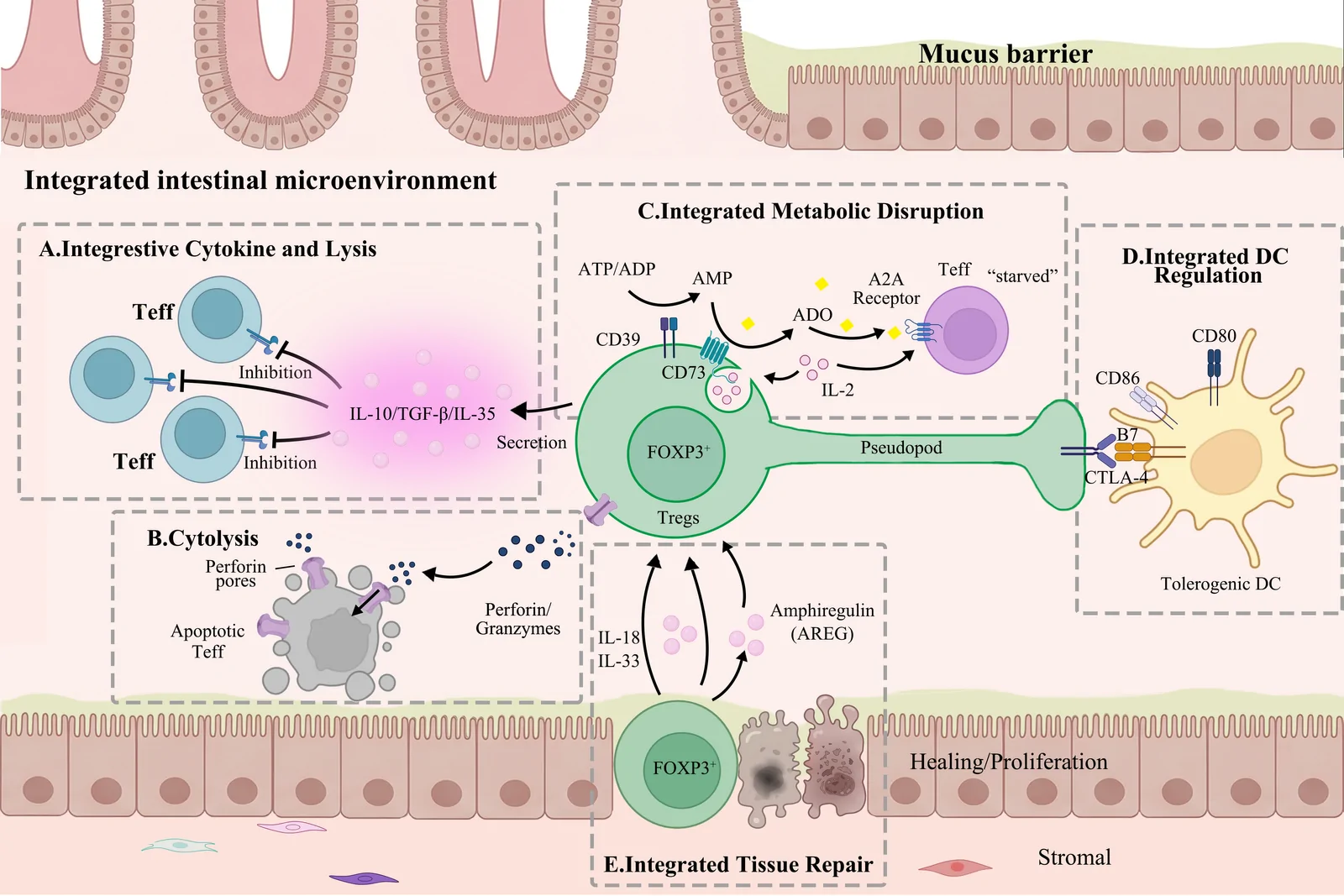
** Functional mechanisms of Tregs in the intestinal microenvironment.** Tregs exert immunosuppressive effects through multiple mechanisms. (A) Tregs secrete anti-inflammatory cytokines (IL-10, TGF-β, and IL-35) to inhibit effector T cells (Teff), (B) and induce cytolysis of Teff via perforin/granzyme-mediated pathways. (C) Tregs express CD39 and CD73 to convert ATP into adenosine (ADO), which induces metabolic disruption in Teff through A2A receptor signaling. (D) Tregs interact with dendritic cells (DCs) via CTLA-4 binding to CD80/CD86, promoting the generation of tolerogenic DCs. (E) In response to IL-18 and IL-33, Tregs secrete AREG to promote intestinal epithelial healing and proliferation.

Tregs employ multiple immunosuppressive mechanisms, which can be broadly classified into four pathways: cytokine secretion, cytolysis, metabolic disruption, and dendritic cell (DC) modulation. Additionally, emerging evidence indicates that Tregs, independently of their suppressive functions, possess direct tissue repair capacity (Figure 2).

### 3.1 Cytokine-mediated inhibition

Tregs inhibit the functions of effector T cells and antigen-presenting cells in a non-contact-dependent manner by secreting cytokines such as IL-10, TGF-β, and IL-35 53, 54. Among these, IL-10 is particularly crucial in regulating intestinal inflammation, Treg-specific IL-10 deficiency alone is sufficient to induce colitis 55.

In addition, bystander inhibition is one of the core mechanisms by which Tregs activated by one antigen can suppress effector T cells of unrelated specificities in the same microenvironment. The IL-2 produced by adjacent effector T cells can activate MBP-specific Tregs and initiate contact-independent inhibition of local effector T cells; at the same time, the direct contact between Tregs and effector cells can enhance the transfer of soluble mediators, amplifying the inhibitory effect 56.

### 3.2 Cytolytic activity

Tregs can directly kill target cells through pathways dependent on perforin and granzyme 57, 58 - including activated CD4⁺ and CD8⁺ T cells, monocytes, and DCs. It is notable that different subsets employ different mechanisms: human adaptive Tregs express granzyme B, whereas natural Tregs express granzyme A, and there are species-specific differences in the requirement for perforin 57.

### 3.3 Metabolic disruption

Tregs interfere with the metabolism and function of effector T cells through three distinct mechanisms. Owing to their constitutive high expression of CD25 (the high-affinity IL-2 receptor), Tregs consume large amounts of IL-2, depriving effector T cells of this essential growth factor and inducing their apoptosis 59-61.

In addition, Tregs co-express CD39 and CD73, a surface signature distinguishing them from other T cells. CD39 converts ATP/ADP to AMP, and CD73 converts AMP to adenosine. Adenosine acts on the A2A adenosine receptor on effector T cells, inhibiting their proliferation and function 62, 63. Interestingly, A2A signaling also promotes TGF-β secretion by suppressing IL-6 expression, thereby enhancing adaptive Tregs generation 64.

Upon co-cultured with effector T cells, Tregs can also form gap junctions through cell contact-dependent mechanisms, transferring the high levels of cAMP present within the natural Tregs (nTregs) to the effector T cells, thereby directly inhibiting the function of the effector T cells 65.

### 3.4 Dendritic cell modulation

Tregs regulate DCs maturation and function through surface molecules such as CTLA-4 and LAG-3, indirectly inhibiting the effector T cells activation. Tregs-expressed CTLA-4 interacts with B7 molecules (CD80/CD86) on DCs, reducing co-stimulatory molecule expression and impairing DC antigen-presenting capacity 66. This maintains DCs in an immature state, sustaining peripheral tolerance under steady-state conditions 67.

In addition to CTLA-4, LAG-3 (lymphocyte activation gene 3) is also an important inhibitory molecule on the surface of Tregs. LAG-3 negatively regulates T cell expansion through its intracellular signaling domain 68.

Beyond surface molecules, Tregs affect DC metabolism. Mouse CD4⁺CD25⁺ Tregs induce DC expression of indoleamine 2,3-dioxygenase (IDO) in a CTLA-4-dependent manner. IDO depletes tryptophan from the local microenvironment, inhibiting T cell proliferation 69. In the presence of bacterial lipopolysaccharide (LPS), Tregs can also induce the expression of IDO through a cytokine-dependent pathway, indicating that Tregs can regulate the immunosuppressive function of DCs routes depending on the 69.

### 3.5 Tissue repair: a suppression-independent function

Beyond immunosuppression, Tregs possess direct tissue repair capacity. This activity is triggered by the inflammatory mediators IL-18 or IL-33—not by TCR signals—and is mediated by amphiregulin (AREG) secretion 70. This suppression-independent function is particularly relevant in the intestine, where Tregs contribute to mucosal healing alongside immune regulation (see Section 4).

Beyond immunosuppression, Tregs possess a direct tissue-reparative function mediated by amphiregulin (AREG) secretion, which is triggered by IL-18 or IL-33 rather than TCR signals 70. In a murine model of influenza-induced lung injury, selective ablation of AREG in Tregs led to exacerbated tissue destruction, while viral clearance and T-cell responses remained intact—indicating that AREG-driven repair operates through a pathway distinct from classical Treg-mediated suppression 70. Similarly, IL-33/ST2 signaling has been shown to support Treg accumulation and function during intestinal inflammation 71, and engineered human ST2+ Tregs upregulate AREG upon IL-33 stimulation independently of TCR engagement 72.

Nevertheless, it should be noted that the evidence for AREG-mediated tissue repair has been demonstrated predominantly in experimental acute injury models. In chronic autoimmune conditions, such as type 1 diabetes and experimental autoimmune encephalomyelitis, AREG deficiency exerts no significantly impact on disease progression, suggesting a context-dependent role 73. Importantly, while AREG production can be induced independently of TCR stimulation, TCR-dependent signals remain essential for Treg activation, tissue localization, expansion, survival, and classical immunosuppressive function.

## 4. Intestinal microenvironmental shaping of Tregs fitness

The gut imposes unique regulatory layers on Tregs—trillions of commensals, their metabolites, and continuous dietary antigens—shaping tissue-specific phenotypes and functions distinct from systemic Tregs.

### 4.1 Microbiota and metabolite instruction

#### 4.1.1 Symbiotic microbiota and their surface molecules

The interaction between the intestinal microbiota and Tregs is one of the core mechanisms for maintaining intestinal homeostasis. Previous studies have rationally screened 17 strains of Clostridia from healthy human feces and found that they can promote the expansion of colonic Tregs and the expression of anti-inflammatory molecules such as IL-10 and ICOS by providing bacterial antigens and a TGF-β-rich microenvironment. These strains belong to the Clostridium clusters IV, XIVa, and XVIII, lack toxin genes, and have good safety 74.

The molecular basis was established by Mazmanian et al., who showed that a single capsular polysaccharide (PSA) from *Bacteroides fragilis* induces IL-10-producing CD4⁺ T cells and protects against colitis 75. This paradigm was later validated in humans: PSA directly induces FOXP3⁺ Tregs from human naïve CD4⁺ T cells *in vitro*, and IBD patients exhibit reduced PSA expression due to phage-driven promoter inversion, correlating with diminished intestinal Tregs. Other commensals, such as cell surface galactan (CSGG) from *Bifidobacterium bifidum*, also induce Tregs 76. Importantly, this mechanism extends to fungi. Yeast cell wall mannan/β-glucan (MGCP) drives Tregs differentiation via the Dectin-1-Cox2 axis in dendritic cells, while simultaneously inhibiting Th1 IFN-γ through a TLR2-dependent mechanism 77.

#### 4.1.2 Dietary metabolites

Among the numerous metabolites produced by the gut microbiota, short-chain fatty acids (SCFAs) and bile acid metabolites are two core molecules that regulate regulatory T cells (Tregs). The regulatory mechanism of SCFAs on Tregs has a multi-pathway and multi-target feature.

Colonic butyrate concentration correlates positively with Treg abundance. Butyrate facilitates Treg differentiation through several non-exclusive mechanisms. On one hand, it can act directly on naïve T cells under Treg-polarizing conditions to enhance histone acetylation at the FOXP3 promoter and conserved non-coding sequences, thereby enhancing FOXP3 transcription 78, 79. In parallel, butyrate acts indirectly by engaging GPR109A on colonic dendritic cells and macrophages, thereby promoting an anti-inflammatory phenotype that favors Treg differentiation. Additionally, it can modulate intestinal epithelial cells to create a tolerogenic microenvironment 80, 81. Collectively, these direct and indirect pathways form a comprehensive regulatory network linking microbial metabolites to intestinal immune homeostasis.

In terms of bile acid metabolites, studies have identified two lithocholic acid derivatives, 3-oxoLCA and isoalloLCA, regulate the Th17/Tregs balance: 3-oxoLCA directly binds RORγt to block Th17 differentiation, while isoalloLCA promotes FOXP3 expression via the CNS3 enhancer 27. Secondary bile acids also activate the TGR5 receptor on DCs, triggering cAMP-PKA signaling that inhibits NF-κB and pro-inflammatory cytokines, thereby fostering immune tolerance 82.

The clinical relevance of these pathways in IBD was confirmed by multi-omics: fecal SCFA levels are reduced, and bile acid profiles are altered (e.g., decreased secondary bile acids) 83. Additionally, dietary vitamin A metabolite retinoic acid, produced by intestinal DCs via RALDH2, synergizes with TGF-β to induce pTregs differentiation 41. This mechanism complements the regulation of Tregs by microbial metabolites, jointly forming a complex regulatory network of diet - microbiota - metabolites - Tregs.

### 4.2 Specialized intestinal Tregs subsets

Specific bacterial species in the human intestinal symbiotic microbiota can induce the generation of RORγt⁺ Tregs in the colon of mice. Up to 40-60% of colonic Tregs co-express FOXP3 and RORγt, a subset nearly absent in spleen and lymph nodes 84. These Helios⁻Nrp1⁻ pTregs depend on microbiota colonization. Notably, the transcriptional footprint of RORγt in Tregs is completely distinct from that in Th17 cells 84, 85. Mechanistically, RORγt stabilizes FOXP3 by repressing T-bet 30 and drives IL-10 production via c-MAF 31. RORγt⁺ Tregs play a bidirectional role in type 2 immunity: their absence enhances anti-helminth defense but also exacerbates Th2 pathology 86. Their induction requires RORγt⁺ antigen-presenting cells (possibly type 3 innate lymphoid cells or Janus cells) expressing MHCII, CCR7, and αv integrin; loss of these factors redirects T cells toward pathogenic Th17 87.

Most intestinal GATA3⁺ Tregs are Helios⁺ (tTregs origin) and are microbiota-independent 84. They express ST2, the IL-33 receptor, and respond to epithelial-derived IL-33 alarmins. IL-33, together with TCR signals and IL-2, promotes GATA3 upregulation, FOXP3 and ST2 expression, and Tregs proliferation. GATA3 and FOXP3 form a complex after TCR stimulation, enhancing Tregs stability and accumulation in the gut 32, 88.

RORγt⁺ and GATA3⁺ Tregs are mutually exclusive, responding to microbial signals and tissue damage signals, respectively, and their complementary functions maintain intestinal homeostasis.

### 4.3 Intestinal homing and localization

Tregs homing to the gut begins in the gut-associated lymphoid tissues, particularly the mesenteric lymph nodes (mLNs), and culminates in stable residence within the lamina propria. Different intestinal segments drain to distinct mLNs with non-redundant functions: proximal small intestine-draining mLNs favor pTregs induction, whereas distal mLNs drive pro-inflammatory responses 89. After priming in mLNs, Tregs upregulate gut-homing receptors (CCR9, integrin α4β7) in response to retinoic acid and TGF-β produced by CD103⁺ DCs 41, 42, 90.

Single-cell RNA sequencing and pseudo time analysis reveals a dynamic trajectory: Tregs upregulate tissue-homing genes (*Itgae*/Cd103) in mLNs and, upon entering the colonic lamina propria, upregulate effector markers (*Il10*, *Gzmh*, *Areg*) while downregulating lymph node-homing markers (*Ccr7*, *Sell*) 91.

Long-term Tregs residence in the intestine requires integration into a stable cellular niche. The lamina propria, not lymphoid aggregates, is the primary site sustaining effector Tregs function, where CD206⁺ macrophages form stable spatial interactions with Tregs to maintain tolerogenic capacity 92. This discovery fills a critical gap in understanding how Tregs achieve durable residence after homing.

**Figure 3:**
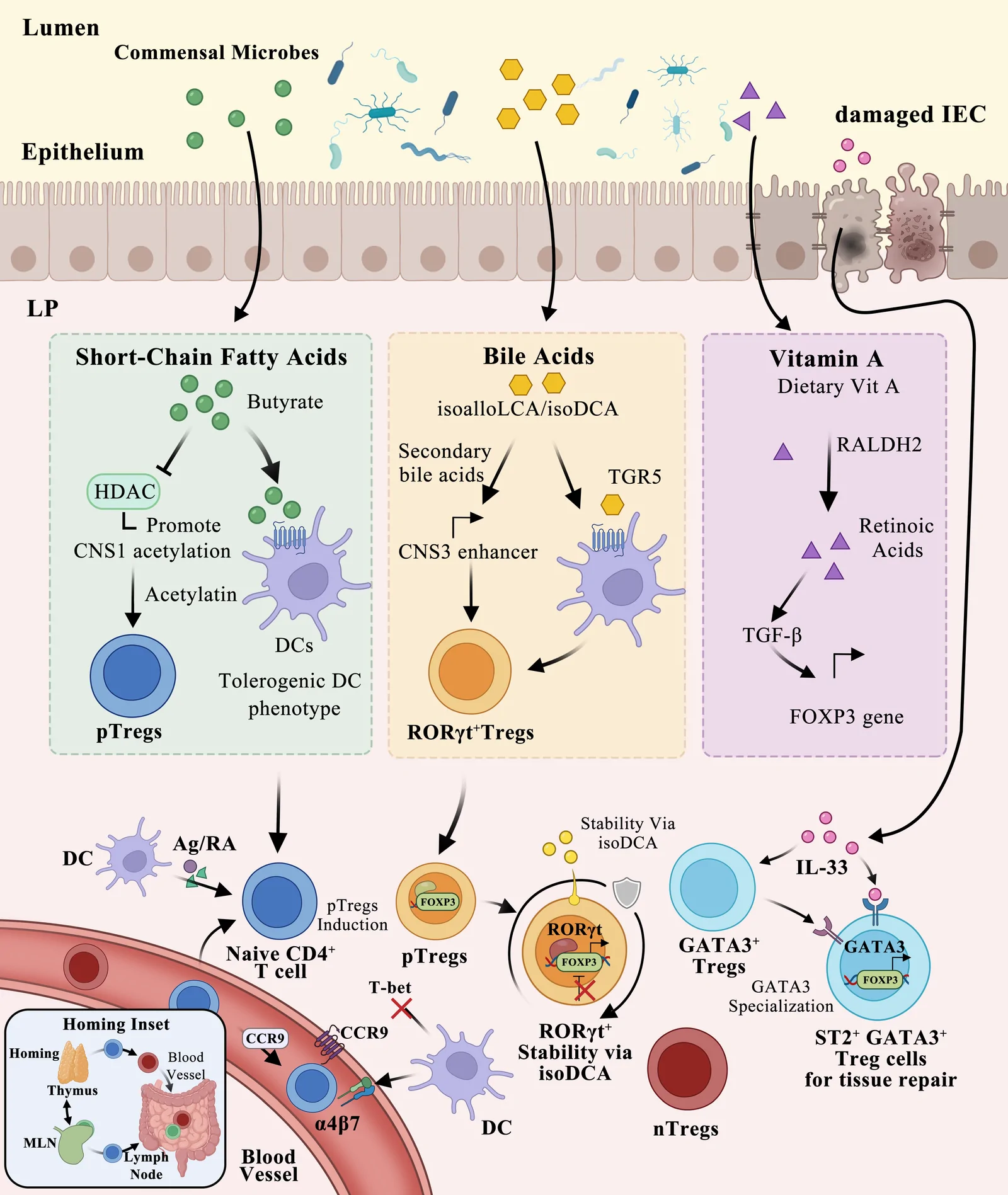
** Intestinal microenvironmental regulation of Tregs differentiation and function.** In the lamina propria (LP), naïve CD4⁺ T cells interact with dendritic cells (DCs) presenting antigen and retinoic acid (RA), promotes pTregs induction, differentiation via dependent FOXP3 expression and stabilizes RORγt⁺ Tregs. Commensal Clostridia produce butyrate and isoDCA, which promote pTregs differentiation and stabilize RORγt⁺ Tregs, respectively. Gut-homing receptors CCR9 and α4β7 direct Tregs to the small intestine. Concurrently, damaged intestinal epithelial cells (IECs) release IL-33, which drives GATA3⁺ ST2⁺ Tregs specialization.

Through coordinated microbial signals, dietary metabolites, and homing cues, the intestinal environment shapes specialized Tregs subsets that preserve immune homeostasis (Figure 3).

## 5. Treg dysfunction in intestinal pathologies

### 5.1 IBD: FOXP3 instability hypothesis

The intestinal Treg dysfunction in IBD reflects not a simple numerical deficit but a qualitative collapse of FOXP3 protein stability driven by a convergence of inflammatory cytokines and dysregulated metabolites. This section presents the mechanistic evidence supporting what we term the FOXP3 instability hypothesis —a unifying framework that explains why expanded Tregs often fail to control inflammation and points toward a new therapeutic logic: stabilizing FOXP3 protein, rather than merely increasing Treg numbers.

#### 5.1.1 Inflammatory cytokines attack the FOXP3 stability network

TNF-α and IL-6, both elevated in the IBD mucosa, directly impair FOXP3 expression. TNF-α induces endoplasmic reticulum (ER) stress in Tregs via the IRE1α-p38 signaling axis, leading to FOXP3 downregulation. The E3 ligase Hrd1 normally antagonizes this ER stress response to preserve Tregs stability, but when overwhelmed by sustained inflammation, Tregs become destabilized. Meanwhile, IL-6 drives Tregs toward a Th17-like phenotype in the presence of TGF-β, generating exFOXP3 Th17 cells with heightened pathogenicity. Thus, the cytokine-rich IBD environment actively erodes FOXP3 expression through both ER stress-dependent and lineage-plasticity pathways 44. It should be noted that the mechanistic insights outlined above are based chiefly on murine models and in vitro experimental systems; consequently, their direct applicability to human IBD remains to be further established.

#### 5.1.2 Succinate: a pathogenic metabolite that degrades FOXP3

Beyond cytokine signals, the disrupted metabolic landscape in IBD delivers an additional blow directly to FOXP3 protein integrity. Wang et al. (2025) recently demonstrated that succinate levels are markedly elevated in the colonic mucosa of IBD patients and mouse models, and this elevation is pathogenic 46. Mechanistically, succinate selectively reduces the expression of 2-oxoglutarate dehydrogenase complex (OGDHc) — the enzyme that converts α-ketoglutarate to succinyl-CoA—which in turn diminishes FOXP3 succinylation. The loss of this succinylation modification exposes FOXP3 lysine residues to the ubiquitin-proteasome system, leading to FOXP3 protein degradation. Genetic deletion of *Dlst* (encoding a core OGDHc component) in Tregs recapitulates this phenotype, causing reduced FOXP3, impaired suppressive function, and severe gut inflammation—all fully rescued by restoring FOXP3 expression. In human IBD, FOXP3 and OGDHc levels in Tregs are reduced and negatively correlate with succinate levels and disease severity. This succinate-OGDHc-FOXP3 axis thus constitutes a molecular switch from protective succinylation to pathogenic ubiquitination, directly linking a disease-elevated metabolite to Tregs functional collapse 46. It should be noted that this axis has been characterized mainly in mouse colitis models and human cell lines; its prospective validation in human IBD cohorts is therefore warranted.

However, it should be noted that the succinate-OGDHC-FOXP3 axis has been primarily characterized by a single research group to date 46, and independent corroboration from other laboratories is still warranted. Accordingly, while this mechanism offers a compelling molecular rationale for Treg dysfunction in IBD, it should currently be regarded as a promising hypothesis pending further validation by additional independent studies.

#### 5.1.3 Protective metabolites vs. disruptive signals: a two-faced environment

The same intestinal milieu that in health promotes Tregs stability through short-chain fatty acids (SCFAs), bile acid metabolites (e.g., isoalloLCA) 83, and retinoic acid becomes, in IBD, a source of FOXP3-destabilizing factors. Importantly, the stability-adaptation paradox operates through a shared molecular language: SCFAs enhance FOXP3 expression via histone acetylation and HDAC inhibition, whereas succinate suppresses FOXP3 succinylation to trigger ubiquitination. The net outcome—stable or unstable FOXP3—is determined by the balance between these competing signals. In IBD, this balance tips decisively toward degradation.

#### 5.1.4 When stability mechanisms fail: the exFOXP3 Th17 threat

The failure of internal stability mechanisms leads not merely to loss of suppression but to active lineage conversion. Tregs that lose FOXP3 in the inflammatory environment acquire an exFOXP3 Th17 phenotype, characterized by IL-17A and IFN-γ production, and accumulate at sites of inflammation. In the IBD intestine, where IL-6 and succinate converge, this transformation likely contributes to the breakdown of tolerance and perpetuation of chronic inflammation. Importantly, the compensatory expansion of IL-10-producing Tr1 cells observed in FOXP3^+^ Treg-specific IL-10 knockout mice suggests that the immune system attempts to fill the regulatory niche, but this compensation is itself impaired by ER stress, further compromising resilience 93.

**Figure 4:**
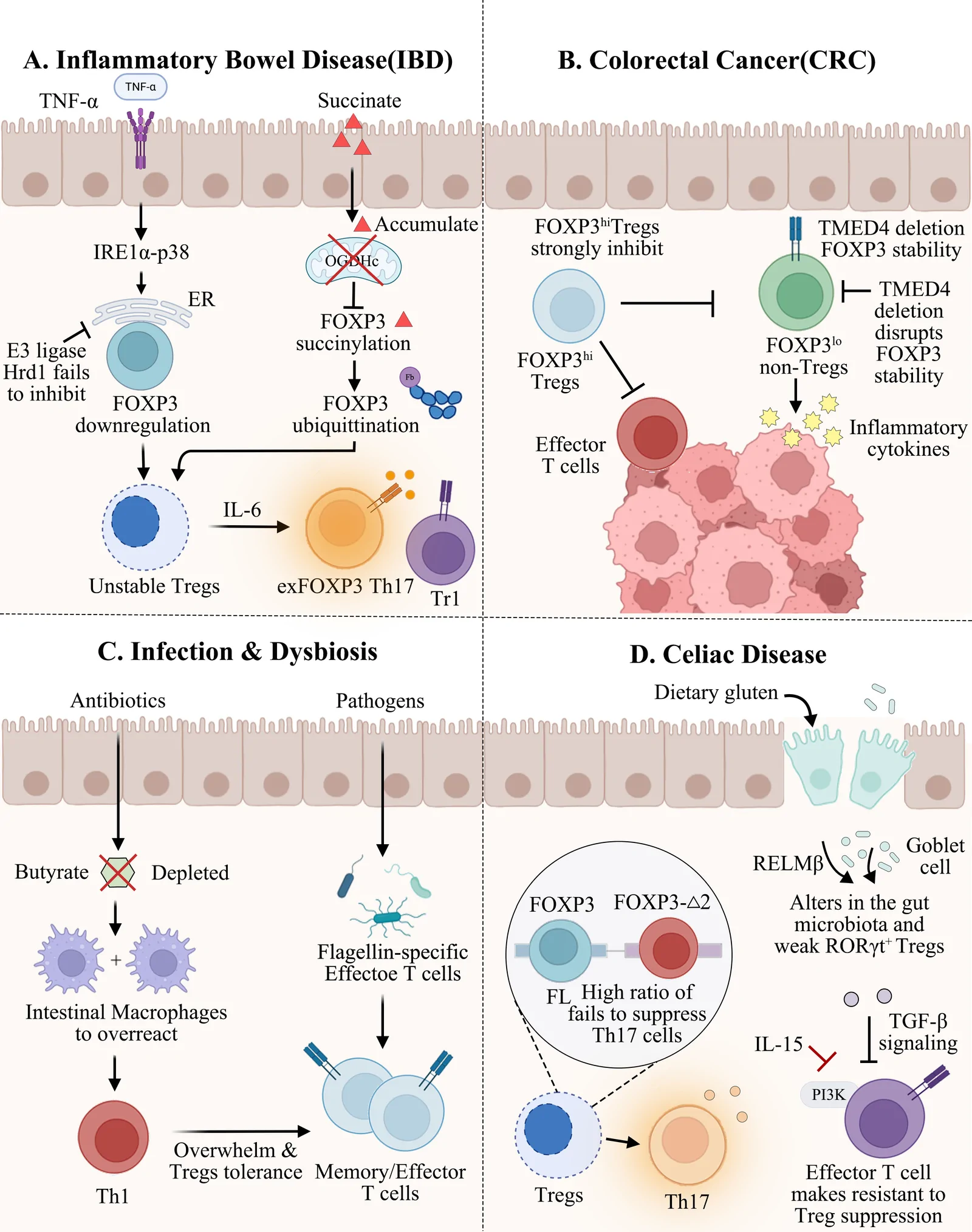
** Treg dysfunction in intestinal pathologies.** (A) In IBD, TNF-α activates IRE1α-p38 signaling, inducing ER stress. The E3 ligase Hrd1 normally antagonizes this ER stress to preserve FOXP3 stability; however, chronic inflammation overwhelms this protective mechanism, leading to FOXP3 downregulation. Accumulated succinate inhibits OGDHc, causing FOXP3 succinylation and ubiquitination, resulting in unstable Tregs conversion to exFOXP3 Th17 cells. (B) In CRC, FOXP3^hi^ Tregs strongly inhibit effector T cells, while TMED4 deletion disrupts FOXP3 stability, promoting inflammatory cytokine production. (C) In infection and dysbiosis, antibiotic-induced butyrate depletion impairs intestinal macrophage function, and pathogen-derived flagellin activates effector T cells that overwhelm Tregs tolerance. (D) In celiac disease, dietary gluten alters gut microbiota and weakens RORγt⁺ Tregs. FOXP3-Δ2 variant (high Δ2/FL ratio) fails to suppress Th17 cells, while IL-15-activated effector T cells become resistant to Tregs suppression.

Implications for the stability-adaptation framework. The IBD evidence directly validates the core premise of our proposed framework: Treg dysfunction is primarily a problem of FOXP3 protein stability, not numerical deficiency. This explains why low-dose IL-2 therapy, while expanding Tregs, shows heterogeneous clinical responses—expanded Tregs remain unstable in the succinate-rich, cytokine-laden IBD microenvironment 55. It also identifies specific therapeutic nodes: blocking succinate-induced degradation (via OGDHc activation or enhancing Hrd1/TMED4 cytoprotective pathways) or reinforcing FOXP3 succinylation may offer more rational strategies than non-selective Tregs expansion 45, 94. Figure 4 illustrates the convergence of two major FOXP3-destabilizing pathways in IBD: the succinate-OGDHc axis, which shifts FOXP3 from protective succinylation to pathogenic ubiquitination 95, 96, alongside the ER stress pathways that converge on the same endpoint of FOXP3 instability 97.

The FOXP3 instability hypothesis provides a unifying framework for Treg dysfunction in IBD, yet it does not fully account for all clinical observations. Notably, some IBD patients exhibit normal or even elevated Treg frequencies in peripheral blood and intestinal mucosa yet still develop active disease 98. This phenomenon indicates that Treg quantity does not equate to functional quality—a principle central to the FOXP3 instability hypothesis—but also suggests that additional layers of Treg dysfunction may exist beyond FOXP3 protein degradation. These include impaired Treg trafficking to inflamed tissues 99, reduced expression of key suppressive molecules (e.g., IL-10, CTLA-4) despite intact FOXP3 expression 100, resistance of effector T cells to Treg-mediated suppression, and dysfunction of non-FOXP3 regulatory populations such as Tr1 cells. Furthermore, the relative contribution of succinate-driven FOXP3 degradation versus cytokine-induced (TNF-α, IL-6) FOXP3 instability may vary across IBD subtypes and disease stages 46, a heterogeneity that the current hypothesis does not fully address. Thus, while the FOXP3 instability hypothesis captures a critical pathogenic mechanism, it should be viewed as one component of a multifactorial model of Treg dysfunction in IBD.

### 5.2 Colorectal cancer: temporal duality of immune surveillance and Tregs as therapeutic targets

The regulation of Tregs in colorectal cancer (CRC) exhibits a time-dependent dual role that reflects the evolving nature of the tumor immune microenvironment.

#### 5.2.1 Protective role in the pre-malignant stage

In the pre-malignant stage, particularly in IBD-associated colon carcinogenesis, Tregs exert a protective function by suppressing chronic inflammation—a well-established driver of tumor initiation. Studies have shown that CEA-specific CAR-Tregs can ameliorate colonic inflammation, reduce the severity of colitis, and subsequently decrease the subsequent tumor burden 101. This protective effect aligns with the observation that GPR109A signaling, which promotes Tregs differentiation, suppresses colonic inflammation and carcinogenesis 80. Thus, during the “inflammation-to-cancer” transition, Tregs act as gatekeepers that limit tumor initiation.

#### 5.2.2 Detrimental role in established CRC

Once CRC is established, however, the functional landscape shifts dramatically. Tumor-infiltrating Tregs (TI-Tregs) accumulate in the tumor microenvironment (TME) and actively promote immune evasion. These TI-Tregs exhibit enhanced suppressive capacity, driven by factors such as TGF-β, IL-10, and adenosine, and effectively inhibit anti-tumor effector T cells (CD8⁺ CTLs and Th1 cells). Specific deletion of TMED4—which destabilizes FOXP3 expression—enhances anti-tumor immunity, indicating that Tregs stability is a critical barrier to effective CRC immunotherapy 45.

Importantly, the enhanced antitumor immunity observed upon TMED4 deletion in Tregs reflects the same mechanistic principle underlying TMED4 deficiency-induced colitis exacerbation: loss of TMED4 compromises Treg suppressive function through IRE1α-dependent ROS accumulation and reduced Foxp3 stability 45. The divergent phenotypes are not attributable to tissue- or context-specific roles of TMED4, but rather to the opposing impact of impaired Treg in different pathological contexts—beneficial when it unleashes antitumor immunity, but detrimental when it unleashes autoinflammation in IBD. This duality highlights both the therapeutic potential of targeting TMED4 in cancer immunotherapy and the potential risk of exacerbating autoimmunity, underscoring the need for careful, disease-context-tailored therapeutic approaches.

#### 5.2.3 Heterogeneity of TI-Tregs as a prognostic classifier

Importantly, not all FOXP3⁺ cells in CRC are functionally equivalent. Single-cell studies have shown that FOXP3⁺ cells can be classified into functionally distinct subtypes: FOXP3^h^ⁱTregs cells have complete inhibitory ability and are associated with a poorer prognosis; while FOXP3^lo^ non-Tregs cells without expressing CD45RA and unstable FOXP3 and secrete inflammatory cytokines like IL-17/IFN-γ, which are associated with a favorable outcome 102. This finding indicates that using FOXP3⁺ cells alone as a marker may lead to incorrect prognostic classification and a more precise immunotherapeutic approach may involve strategies that selectively deplete or destabilize FOXP3ʰⁱ Tregs while leaving FOXP3ˡᵒ cells intact.

#### 5.2.4 Tregs as therapeutic targets in CRC

Several Tregs-directed strategies have been explored in preclinical and clinical settings for CRC. These can be broadly categorized into depletion, functional modulation, and combination with immune checkpoint inhibitors. Table 2 summarizes the key approaches, their mechanisms, developmental status, and major challenges.

Notably, emerging evidence suggests that subset-selective depletion (e.g., targeting FOXP3ʰⁱ Tregs via CCR4 or GITR) and metabolic reprogramming (e.g., adenosine pathway blockade) offer better therapeutic windows than global Tregs ablation. Furthermore, combining Tregs-targeted agents with anti-PD-1 therapy has shown synergistic anti-tumor immunity in preclinical CRC models, warranting clinical investigation.

#### 5.2.5 Clinical translation considerations

Despite promising preclinical data, several barriers must be addressed before Tregs-targeted therapies can be translated into CRC clinical practice.

A major challenge is on-target off-tumor toxicity. Systemic Tregs depletion inevitably disrupts peripheral tolerance and can trigger autoimmune manifestations. To mitigate this, local delivery strategies—such as intratumoral injection, prodrug-conjugated antibodies, or nanoparticle formulations—are being explored to confine Tregs depletion to the tumor microenvironment.

Another challenge arises from the functional heterogeneity of tumor-infiltrating FOXP3⁺ cells. As discussed above, FOXP3ˡᵒ non-Tregs may even correlate with favorable prognosis, whereas FOXP3ʰⁱ Tregs are the primary immunosuppressive subset. Therefore, subset-selective approaches—for instance, targeting CCR4 or GITR—are required to avoid eliminating non-suppressive or pro-inflammatory FOXP3⁺ cells.

Daclizumab and Basiliximab are listed in Table 2 as representative CD25-targeting agents, but they were originally developed as immunosuppressive drugs to prevent graft rejection by blocking IL-2 binding to CD25 on effector T cells, which also impairs Teff activation and antitumor immunity 103. These first-generation anti-CD25 antibodies showed no clinical benefit in cancer and were consequently dropped from oncology development; no dedicated Phase II/III trials in colorectal cancer have been reported. Newer non-IL-2-blocking anti-CD25 antibodies (e.g., RG6292) are designed to deplete Tregs while preserving IL-2 signaling on Teffs, a strategy considered more promising for cancer immunotherapy 104. We have therefore revised the development stage in the table to “Discontinued in oncology” to better reflect the evidence.

A further consideration involves compensatory Tregs regeneration. Following systemic depletion, peripheral induced Tregs (pTregs) can rapidly repopulate the suppressive niche, often within days. Combining Tregs depletion with IL-2 blockade or mTOR inhibition may delay or prevent this rebound, a strategy that warrants further preclinical optimization.

Finally, the lack of predictive biomarkers represents a critical bottleneck for patient stratification. Currently, no validated marker exists to identify which CRC patients are most likely to benefit from Tregs-directed therapies. Candidate biomarkers include the FOXP3ʰⁱ/CD8⁺ ratio in tumor biopsies, TCR clonality analysis, or serum levels of soluble factors such as IL-10 or adenosine. Prospective validation in well-designed clinical trials is urgently needed.

### 5.3 Infection and dysbiosis

Intestinal immune homeostasis depends on intact microbiota-Tregs crosstalk. Infection or antibiotic treatment disrupts this axis, leading to Tregs numerical loss, functional impairment, or lineage instability.

Acute gastrointestinal infections activate not only pathogen-specific immunity but also microbiota-specific T cells (e.g., flagellin-specific T cells), which differentiate into effectors and form memory cells 105. This effector expansion can override Tregs-mediated tolerance to commensal antigens.

Antibiotic treatment depletes butyrate, leading to excessive activation of intestinal macrophages, persistent Th1 responses, and microbial dysbiosis. 81. Given that butyrate is essential for Tregs induction and maintenance, antibiotics likely disrupt the microbial community-SCFAs-Tregs regulatory axis, compromising immune homeostasis.

### 5.4 Celiac disease

Celiac disease is a chronic small intestinal inflammatory disorder triggered by dietary gluten, driven by disruption of oral tolerance. The dysfunction of Tregs plays a crucial role in this process.

In active celiac disease, intestinal biopsies show a significantly higher ratio of the FOXP3Δ2 splicing isoform relative to the full-length isoform, whereas healthy controls maintain balanced expression. FOXP3Δ2 lacks exon 2 and fails to effectively suppress Th17-driven immune responses 106. This demonstration links alternative FOXP3 splicing to celiac disease pathogenesis.

The cytokines in the intestinal microenvironment further impair the function of Tregs. IL-15 is significantly overexpressed in the intestines of patients with celiac disease, which weakens the activity of Tregs by disrupting TGF-β signaling and, via PI3K activation, renders effector T cells resistant to Tregs-mediated suppression 107. Healthy individuals maintain tolerance to dietary gluten and commensal fungi through selective expansion of antigen-specific Tregs. In celiac patients, this balance is lost, leading to expansion of pathogenic T cells 108, 109.

The latest research reveals that epithelial factors regulate Tregs through microbial metabolites. Goblet cell-derived RELMβ is highly expressed in food allergies patients and mouse models. By consuming* Lactobacillus* and *Alistipes* that produce indole metabolites, it weakens RORγt^+^ Tregs and disrupts oral tolerance. This study links epithelial factors, microbiota, metabolites and Tregs into a complete regulatory axis 110.

Together, these findings reveal that in celiac disease, Treg dysfunction arises from a combination of intrinsic FOXP3 instability, cytokine-induced resistance, and disrupted microbial-epithelial signaling—all converging on the breakdown of oral tolerance.

## 6. Tregs-targeted therapeutic strategies

### 6.1 Adoptive Tregs cell therapy

Restoring the function of Tregs, rather than merely increasing their numbers, has emerged as a rational strategy for re-establishing intestinal immune tolerance. Initial cell therapy studies-based approaches used autologous hematopoietic stem cell transplantation to induce remission in refractory CD 111-113. Although this strategy offers the promise of immune reset, its non-selective nature carries a high burden of adverse events, prompting a shift toward therapies that target specific immune cell populations rather than the immune system.

Tregs adoptive transfer is a therapeutic strategy for restoring immune tolerance. Adoptive transfer of ex vivo-expanded Tregs has been tested in graft-versus-host disease (GvHD) and type 1 diabetes, establishing safety and feasibility 114, 115. Trzonkowski *et al*. first infused donor-derived in vitro expanded CD4^+^CD25^+^CD127^-^ Tregs into GvHD patients: chronic GvHD improved, but only transient effects were seen in acute severe disease 116. In T1D, two independent phase I trials (Marek-Trzonkowska et al. and Bluestone et al.) confirmed that autologous polyclonal Tregs infusion is safe, with long-term persistence of transferred cells and partial preservation of β-cell function 114, 115.

Similarly, in the clinical studies of IBD, the CATS1 phase I/IIa study used ovalbumin-specific Tregs in refractory CD patients. The treatment was well tolerated, and dose-related clinical responses were observed at 10⁶ cells 117. More recently, patients with refractory ulcerative colitis and concomitant primary sclerosing cholangitis who received autologous polyclonal Tregs infusion achieved sustained clinical, endoscopic and histological improvements for approximately 12 weeks, accompanied by enrichment of FOXP3^+^ Tregs and IL-10^+^ T cells in the intestinal mucosa 118.

In addition to the classic FOXP3^+^ Tregs, type I regulatory T cells (Tr1) are regarded as another ideal candidate for cell therapy due to their clear IL-10 and TGF-β mediated inhibitory functions 53. The clinical-grade product T-allo10, rich in Tr1 cells, has been used in patients receiving HLA-mismatched hematopoietic stem cell transplantation. Tr1 cells express high levels of CTLA-4 and PD-1 and persist in peripheral blood for up to one year post-infusion, providing a mechanistic basis and monitoring tool for Tr1-based immunotherapy 119. Despite these promising results, adoptive Tregs therapy faces a fundamental limitation: Tregs expanded *in vitro* often lose FOXP3 expression and suppressive function after infusion into the inflammatory intestinal milieu. This instability—driven by succinate, TNF-α, and IL-6 as detailed in Section 5.1—explains why some patients show robust Tregs expansion yet fail to achieve clinical improvement. Consequently, optimizing cell manufacturing to generate functionally stable Tregs has become a priority. Hypoxic culture conditions enhance the yield and stability of induced Tregs (iTregs) by upregulating the glucose transporter GLUT1 and increasing FOXP3 expression, without compromising suppressive capacity 120. More recently, proteasome inhibitor-mediated stabilization of the heat shock factor HSF1 has been shown to promote a CD69⁺ iTregs subset with superior efficacy in colitis models 121, 122. These approaches aim not simply to expand Treg numbers but rather to lock FOXP3 expression and functional integrity—embodying the shift from 'more Tregs' to 'stable Tregs' that will be essential for successful clinical translation.

### 6.2 Pharmacological Tregs modulation

Pharmacological agents that expand or stabilize Tregs offer an alternative to adoptive cell transfer. Among these, low-dose IL-2 has been the most extensively studied. However, a fundamental limitation of low-dose IL-2 is its inability to discriminate between Tregs and effector T cells at higher doses, narrowing the therapeutic window. Moreover, more critically for the stability-adaptation paradox—IL-2 expands Tregs quantitatively but does not correct the qualitative instability of FOXP3 in the inflammatory milieu.

To overcome the narrow window of native IL-2, protein engineering has produced Tregs-selective variants. Peterson *et al.* first demonstrated the safety and efficacy of the long-acting Tregs-selective IL-2 mutein, (IgG-(IL-2N88D)₂) in non-human primates and humanized mice expanded Tregs 10- to 14-fold after a single low-dose injection without affecting effector T cells 123.

Based on this, Khoryati et al. further engineered IL-2 mutants with enhanced CD25 dependence. When fused with Fc, these mutants could selectively expand Tregs within a wide dose range while avoiding the activation of effector cells. Due to the reduced receptor-mediated clearance, achieved more persistent Tregs expansion. In the NOD diabetes model, infrequent administration sufficed to arrest disease 124. These engineered muteins represent an advance in Tregs quantity, but they do not directly address FOXP3 stability.

Based on the above preclinical successes, low-dose IL-2 entered clinical testing for IBD. Allegretti et al. conducted a phase Ib/IIa trial in patients with moderate-to-severe ulcerative colitis, demonstrating for the first time that low-dose IL-2 is feasible and expands Tregs in IBD patients. However, the study also revealed marked therapeutic heterogeneity: some patients achieved robust Tregs expansion yet showed no clinical improvement, while others improved despite only modest Tregs increase. This dissociation between Tregs quantity and outcome is precisely what the FOXP3 instability hypothesis predicts—expanded Tregs remain susceptible to succinate-driven degradation and exFOXP3 conversion in the inflamed gut. Thus, while low-dose IL-2 and its engineered derivatives are valuable tools for increasing Tregs numbers, they are unlikely to achieve durable remission unless combined with strategies that actively stabilize FOXP3 protein (e.g., HDAC inhibitors, HSF1 stabilizers, or OGDHc activators). The next generation of pharmacological Tregs modulation must therefore shift from expansion-only to stability-plus-expansion approaches 40.

### 6.3 Engineering Tregs

The antigen non-specificity of polyclonal Tregs therapy limits its efficacy, driving efforts to endow Tregs with antigen specificity via genetic engineering. Currently, the main engineering methods include two technical routes: TCR gene transfer and chimeric antigen receptor (CAR) engineering.

TCR gene transfer endows Tregs with MHC-restricted targeting ability by introducing antigen-specific TCRs. Wright and his team proposed and verified a strategy for preparing engineered Tregs that does not require the isolation of rare natural Tregs clones or knowledge of pathogenic antigens. This strategy involves reprogramming natural Tregs or converting conventional T cells into Tregs through FOXP3^+^TCR co-transduction via TCR gene transfer, enabling them to efficiently home to inflammatory sites under antigen-driven conditions and locally suppress Th17 cells through a chain inhibition mechanism 125. TCR affinity is critical—high-affinity clones confer stronger antigen-specific suppression than low-affinity ones 126. However, not all autoantigen-specific TCRs are equally suitable; for example, islet-specific TCRs show lower reactivity than virus-specific TCRs, necessitating functional screening systems 127-129.

Chimeric antigen receptor (CAR) technology enables MHC-independent recognition of surface antigens, offering reduced IL-2 dependence and enhanced tissue homing 130. Elinav et al. first validated CAR-Tregs in intestinal inflammation using TNP-specific CAR-Tregs, which expanded ex vivo without losing FOXP3 or suppressive function 131, 132. This was extended to carcinoembryonic antigen (CEA): CEA-CAR Tregs suppressed colitis and reduced subsequent colorectal tumor burden, illustrating a strategy to interrupt the “inflammation-cancer” axis 101.

Target selection has diversified: FliC-specific CAR-Tregs (targeting microbial flagellin) promoted epithelial barrier repair 133. More recently, IL-23R-specific CAR-Tregs showed minimal tonic signaling, robust phenotypic stability, and maintained FOXP3 expression under chronic inflammatory conditions 134. In addition, CAR-Tregs technology has been applied to multiple disease models, including multiple sclerosis, vitiligo, Alzheimer's disease, asthma, hemophilia and transplant rejection 135-141, demonstrating the potential of its platform-based application.

TCR-Tregs provide more physiological signaling and potentially better stability but are MHC-restricted and require functional TCR screening. CAR-Tregs are MHC-independent and target-flexible but risk tonic signaling-induced exhaustion. Future directions include developing TCR-like CARs or optimizing CAR structure to mimic natural signals, addressing antigen specificity, phenotypic stability, and in vivo persistence.

### 6.4 Microbiota-based interventions

Specific strains can induce the differentiation of Tregs. Clostridia in healthy human feces can promote the expansion of colonic Tregs and the production of IL-10 by providing bacterial antigens and a microenvironment rich in TGF-β, thereby alleviating colitis 74. Additionally, studies have shown that the Clostridiales microbiota can inhibit food allergies by inducing Tregs to express RORγt. This protective effect depends on the intrinsic MyD88 signaling pathway of Tregs 142.

The efficacy of fecal microbiota transplantation (FMT) in ulcerative colitis has been verified in randomized controlled trials. In 2015, Moayyedi et al. found that a weekly FMT enema for 6 weeks achieved clinical remission in 24% of active UC patients, significantly better than the 5% in the placebo group 143. In a subsequent multicenter, double-blind trial with a high-intensity, multi-donor FMT protocol, Paramsothy et al. reported a 27% remission rate, alongside progressive microbiota enrichment and taxon-specific associations with clinical outcome 144.

These studies established the clinical efficacy and safety of FMT in UC. However, it is important to note that the primary endpoints of these trials were clinical remission rather than Treg quantity or function, and no causal relationship between FMT-induced clinical improvement and Treg induction has been established. Although preclinical studies have demonstrated that specific Clostridia strains can promote colonic Treg expansion 74, 142, and microbial consortia such as GUT-108 can induce IL-10+ Tregs in colitis models 145, the translational relevance of these findings to human FMT is still uncertain. Going forward, including immune monitoring of Treg frequency, phenotype, and function will be essential to clarify whether Treg modulation mediates the therapeutic benefits of FMT.

The emerging bacterial population editing technology provides a new tool for precise intervention. By using phage-derived particles to deliver base editors, a single administration can achieve a 93% editing efficiency for the target genes of Escherichia coli in the mouse intestinal tract, providing a technical foundation for in-situ modification of the bacterial population with Tregs-inducing related genes 146.

**Figure 5:**
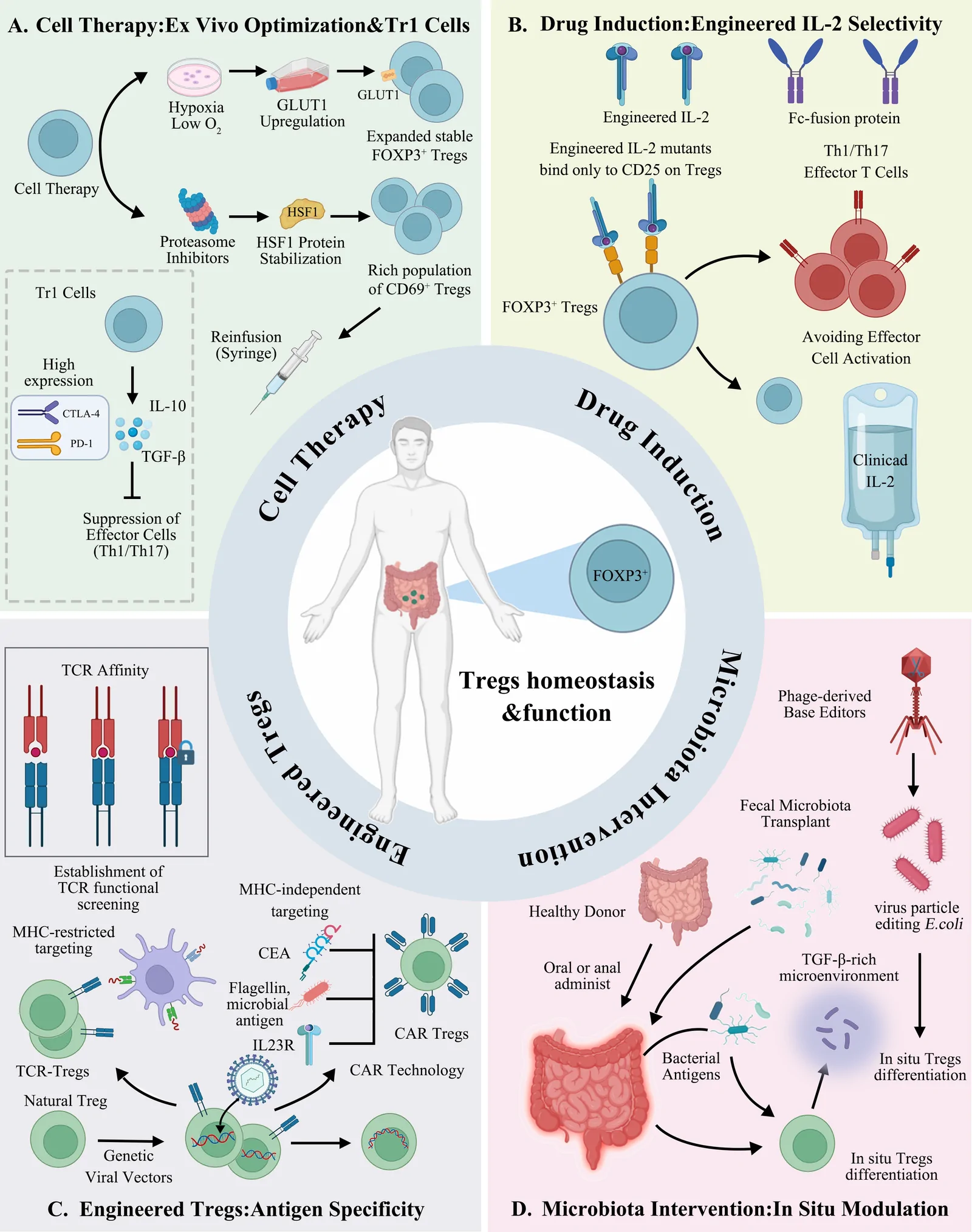
** Tregs-targeted therapeutic strategies for intestinal diseases.** (A) Ex vivo cell therapy: hypoxic culture upregulates GLUT1 to expand stable FOXP3⁺ Tregs; proteasome inhibitors stabilize HSF1 to enrich CD69⁺ iTregs. Tr1 cells suppress effector T cells via IL-10 and TGF-β. (B) Engineered IL-2 mutants selectively bind CD25 on Tregs, expanding FOXP3⁺ cells without activating Th1/Th17 effectors. (C) Antigen-specific engineering: TCR-Tregs enable MHC-restricted targeting; CAR-Tregs (targeting CEA, flagellin, or IL-23R) provide MHC-independent recognition. (D) Microbiota interventions: FMT, synthetic consortia, or phage-delivered base editors reconstruct the gut ecosystem to promote in situ Tregs differentiation.

From strain screening to bacterial consortiums, and then to in-situ gene editing of Escherichia coli, the bacterial population intervention technology is undergoing a development process from transplantation to design and finally to editing 133, 147. Thus, next-generation therapies must shift from more Tregs to stable Tregs. These therapeutic strategies are visually summarized in Figure 5. Additionally, a comparative overview of these strategies with respect to their development stage, advantages, and key challenges is provided in Table 3.

## 7. Limitations, challenges, and risk mitigation

These clinical studies have established the feasibility and safety of Tregs-based therapies, with some patients achieving durable clinical remission. However, translating into routine practice faces significant obstacles.

Tregs instability in the inflammatory niche, tumor immune escape, marked inter-patient variability, and a lack of validated predictive biomarkers 148. Overcoming these challenges is essential to move Tregs-based treatments from proof-of-concept to standard care.

### 7.1 Stability and plasticity risks in inflammatory milieus

The success of Tregs-based therapy not only depends on the survival and homing of the infused cells but critically on their ability to maintain FOXP3 expression and inhibitory function within the inflamed intestinal niche.

#### 7.1.1 The risk of lineage conversion

Preclinical lineage tracing studies have demonstrated that Tregs may lose FOXP3 expression under inflammatory conditions and transform into exFOXP3 effector T cells that produce pro-inflammatory cytokines 96. IL-6, which is elevated in the IBD mucosa, drives Tregs toward a Th17-like phenotype, and these exFOXP3 Th17 cells exhibit enhanced pathogenicity 95, 149. This raises a direct concern for adoptive Tregs therapy: infused cells may not remain stable in the target tissue.

#### 7.1.2 CAR-Tregs Specific instability

Engineered CAR-Tregs face additional stability challenges. The choice of co-stimulatory domain critically influences their functional integrity. Wild-type CD28 signaling domain performs optimally in vivo 147, but CARs also carry an inherent risk of tonic signaling—antigen-independent aggregation of the single-chain variable fragment (scFv). Tonic signaling induces early exhaustion, characterized by upregulation of inhibitory receptors (PD-1, TIM3) and exhaustion-associated transcription factors (TOX, BLIMP1), along with global chromatin remodeling and AP-1 family binding 150.

Moreover, different signal domains have distinct effects on this. The CD28 co-stimulation enhances exhaustion, whereas 4-1BB alleviates exhaustion 151. Paradoxically,4-1BB tonic signaling is more detrimental to Tregs than to conventional T cells 152, underscoring the need for Treg-specific engineering principles.

In response to this limitation, Henschel *et al*. addressed this limitation by constructing a bicistronic vector co-expressing FOXP3 and the CAR. This design enabled CAR-Tregs to maintain stable FOXP3 expression even under inflammatory conditions and in the absence of IL-2. Importantly, it did not induce exhaustion or phenotypic changes and demonstrated superior prevention of rejection and Tregs niche filling in humanized transplantation models 153. This strategy exemplifies the shift from 'more Tregs' to 'stable Tregs' in the engineering space.

### 7.2 Tumor immunosuppression and long-term surveillance

Tregs are double-edged swords in cancer. They suppress inflammation and protect against tumor initiation during the pre-malignant stage but, once a tumor is established, promote immune evasion 45, 80, 102. This time-dependent duality dictates a sequential therapeutic strategy: enhance Tregs function during prevention, but selectively weaken tumor-infiltrating Tregs during treatment.

The same principle applies to long-term safety. In autoimmune disease patients receiving Tregs-stabilizing therapies, the risk of future malignancy must be monitored. Conversely, in colorectal cancer patients, molecules that enforce Tregs stability—such as TMED4—may become therapeutic targets for immunotherapy.

### 7.3 Inter-patient variability and the biomarker deficit

Clinical trials have revealed marked inter-patient variability in response to Tregs-based therapies. Although infused Tregs can persist at the population level for over one year 114, individual differences are substantial and may correlate with age, disease stage, and baseline immune status 115. More importantly, Tregs quantity does not equal functional quality.

The CATS1 study in patients with Crohn's disease found that patients receiving a dose of 10⁶ cells had better outcomes than those receiving higher-dose group, suggesting that there might be an "optimal dose window" for Tregs cell therapy 117. Notably, the specific immune response to ovalbumin correlated with clinical response, hinting that the patient's own immune reaction to the infused Tregs might serve as a predictive marker 117. Similarly, low-dose IL-2 therapy in IBD showed heterogeneous efficacy: some patients achieved robust Tregs expansion but no clinical improvement, reinforcing that restoring Treg numbers is insufficient without functional recovery 40.

These observations underscore an urgent need for validated predictive biomarkers. Candidate approaches include monitoring the restricted TCR repertoire and high CTLA-4/PD-1 expression of Tr1 cells 119, as well as emerging markers such as serum succinate levels, TSDR methylation status, or the FOXP3Δ2/FL isoform ratio. Prospective validation of these biomarkers will be essential to stratify patients and realize precision Tregs immunotherapy.

## 8. Outlook and future directions

Remarkable progress has been made in understanding Tregs biology and harnessing these cells for intestinal disease therapy. Yet, several fundamental questions remain unanswered. Looking ahead, three challenges stand out as the most critical barriers to clinical translation and will likely define the next five years of research.

### 8.1 How can Tregs lineage stability be preserved in the inflamed intestinal niche without compromising safety?

Adoptive Tregs therapy faces a core vulnerability: the inflammatory milieu of IBD actively destabilizes FOXP3 expression, driving Tregs toward pathogenic exFOXP3 Th17 cells. Although ER stress pathways (IRE1α-p38, Hrd1) and metabolic factors (succinate) have been identified as destabilizing signals, we lack pharmacological or genetic tools to reinforce Tregs stability *in situ* without impairing suppressive function or increasing tumor risk. Future efforts should focus on gut-restricted HDAC inhibitors, SCFA mimetics, or CRISPR-based epigenetic editing that locks FOXP3 expression in a pro-tolerant state.

Importantly, Tregs also possess a tissue-repair function that is independent of immunosuppression: they secrete amphiregulin (AREG) and promote repair of muscle, central nervous system, and lung tissues 3. Repair is triggered by IL-18 or IL-33, whereas TCR signals drive inhibition 70. This opens the possibility of selectively enhancing the repair function while preserving inhibitory activity. Critically, any stability-enhancing or repair-boosting strategy must be evaluated for long-term cancer risk, particularly in patients with pre-malignant conditions.

### 8.2 Can we design safe, exhaustion-resistant, and lineage-stable CAR-Tregs for intestinal diseases?

CAR-Tregs offer MHC-independent targeting and enhanced homing, but they suffer from tonic signaling-induced exhaustion and FOXP3 instability. Co-expression of FOXP3 with the CAR improves stability, yet it remains unknown whether such engineered cells maintain long-term function without malignant transformation or loss of identity. The field needs standardized benchmarks for CAR-Tregs quality control, including assays for epigenetic stability, exhaustion markers (PD-1, TOX), and in vivo persistence. Logic-gated CARs (AND/NOT) and safety switches (iCasp9, RQR8) should be rigorously tested in large animal models before first-in-human trials for IBD. Moreover, the optimal co-stimulatory domain (4-1BB versus CD28) may differ between Tregs and conventional T cells, demanding subset-specific engineering principles.

Recent advances illustrate the potential of this platform: AAV-specific capsid CAR-Tregs have demonstrated the ability to simultaneously suppress immune responses against viral capsids and the delivery of transgenes 154, providing a new strategy for immune regulation in gene therapy. Off-the-shelf CAR-T cells have the advantages of ready availability and cost reduction, but they face challenges such as GvHD and host immune rejection. These issues are being addressed through gene editing strategies such as TCR and MHC knockout 155. These engineering principles are directly transferable to CAR-Tregs development.

### 8.3 Which biomarkers will predict response to Tregs-directed therapies?

Clinical trials of low-dose IL-2 and adoptive Tregs transfer have revealed marked inter-patient variability: some individuals achieve robust Tregs expansion yet show no clinical improvement, indicating that Tregs quantity does not equal functional quality. The field urgently needs validated predictive biomarkers to stratify patients and guide treatment decisions. Candidate markers include the FOXP3ʰⁱ/CD8⁺ ratio in intestinal biopsies, serum succinate levels (as a proxy for FOXP3 instability), Treg-specific epigenetic signatures (e.g., TSDR methylation status), or the ratio of FOXP3Δ2 to full-length FOXP3 isoforms.

Notably, fecal microbiota transplantation (FMT) offers a fundamentally different approach by reconstructing the entire intestinal ecosystem, and its efficacy is influenced by donor specificity and disease duration 143, providing clues for personalized microbiota intervention strategies. Emerging technologies enable precise *in situ* modification of intestinal bacteria: phage-derived particles successfully deliver base editors, achieving efficient gene editing in intestinal *Escherichia coli* 146. This method establishes the feasibility of directly modifying bacterial functions to enhance Tregs induction, and could eventually yield novel biomarkers or therapeutic targets.

Prospective, multi-center studies are required to qualify these biomarkers for routine use. Until such markers are available, Tregs-based therapies will remain empiric and their full potential unrealized. The stability-adaptation paradox underlies both therapeutic failure and safety concerns.

Looking forward, five key questions must be addressed to translate Tregs-based therapies into clinical reality for intestinal diseases: (1) how to stabilize FOXP3 in inflamed tissues without compromising antitumor immunity; (2) how to map and exploit the spatial and functional heterogeneity of intestinal Treg subsets; (3) how to design next-generation CAR-Tregs with lineage fidelity, exhaustion resistance, and safety switches; (4) how to harness microbiota-derived metabolites as stage-specific Tregs modulators; and (5) which biomarkers can predict patient responses to Tregs-directed therapies. Solving these questions will likely define the next era of precision immunotherapy for the gut.

**Figure 6:**
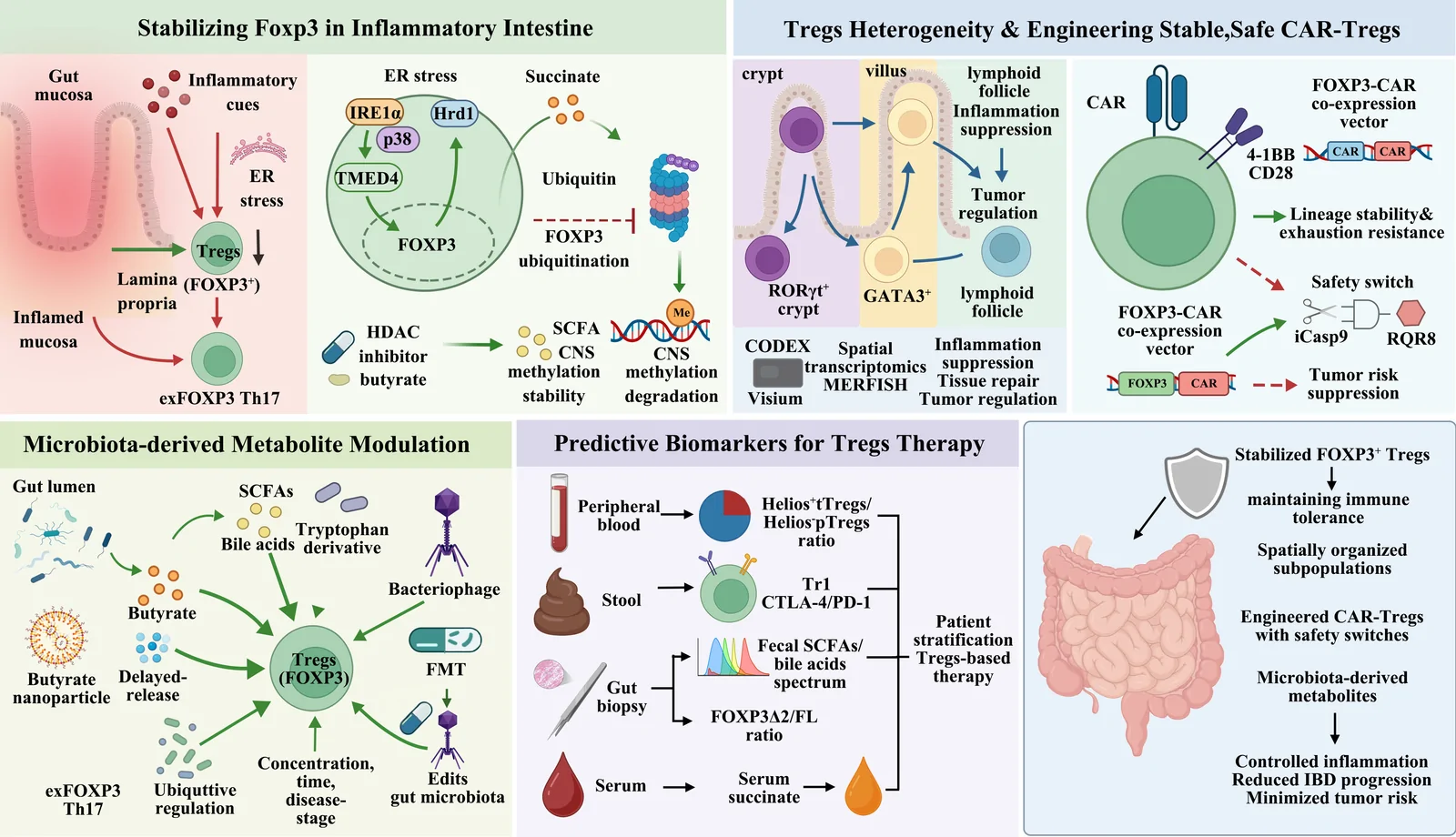
** Future directions for Tregs-based precision immunotherapy in intestinal diseases.** In the inflamed gut, FOXP3 stability is threatened by ER stress and metabolic cues; pharmacological or genetic strategies aim to lock FOXP3 expression and prevent exFOXP3 Th17 conversion. Intestinal Tregs subsets exhibit spatial and functional heterogeneity; next-generation CAR-Tregs require FOXP3 co-expression and safety switches (iCasp9, RQR8) for lineage fidelity. Microbiota-derived metabolites (SCFAs, bile acids, tryptophan derivatives) modulate Treg function. Predictive biomarkers—including blood Helios⁺/Helios⁻ ratio, biopsy FOXP3Δ2/full-length ratio, and serum succinate—are needed to stratify patients. The goal is to establish stabilized FOXP3⁺ Treg populations and safe CAR-Tregs within the gut, enabling precision immunotherapy for intestinal diseases.

**Figure 7:**
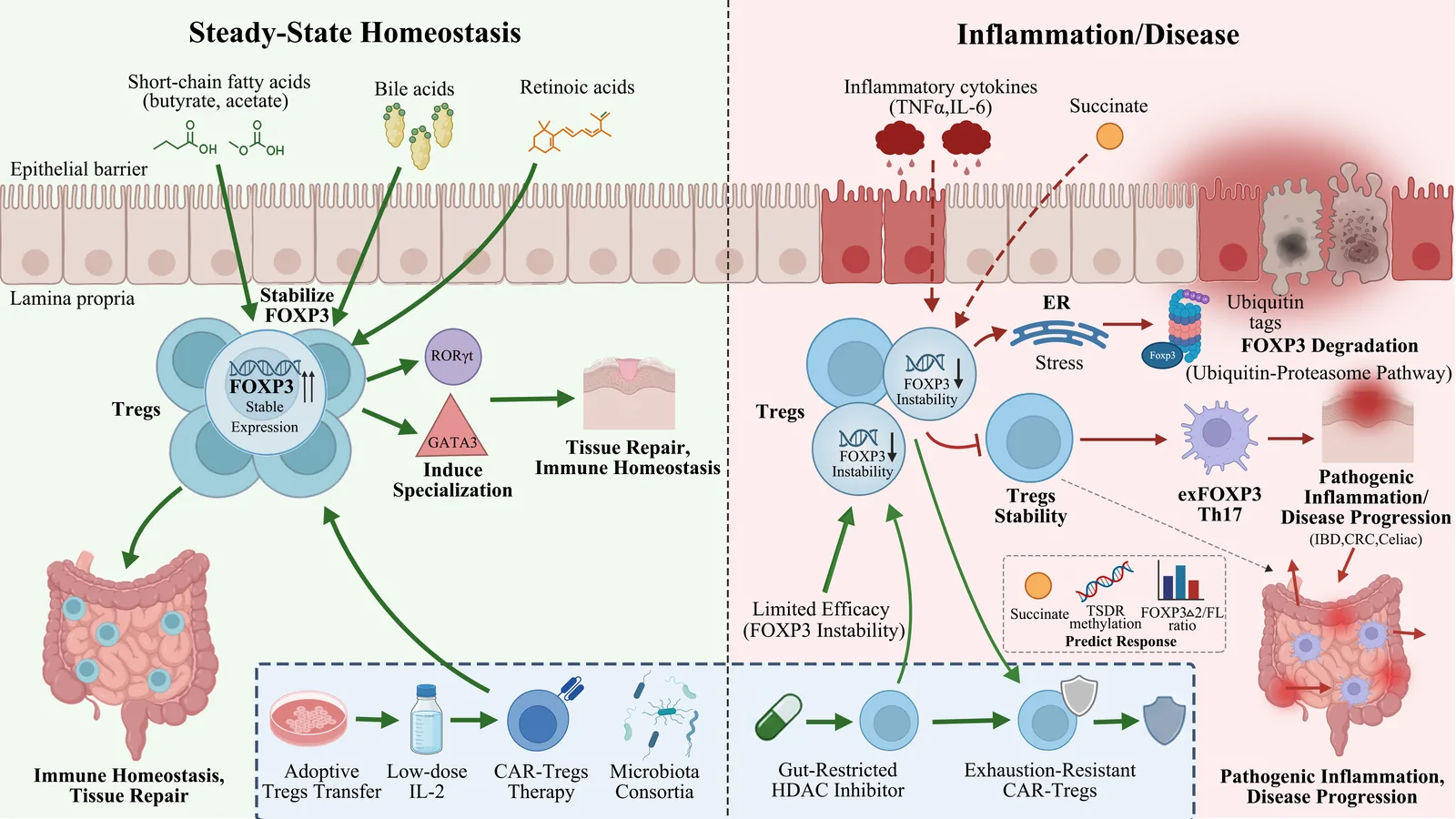
** The stability-adaptation paradox in intestinal Treg biology.** In the steady state (left), microbial metabolites (SCFAs, bile acids) and retinoic acid stabilize FOXP3 and induce RORγt⁺/GATA3⁺ specialization, promoting tissue repair and immune homeostasis. Current therapies (adoptive Treg transfer, low-dose IL-2, CAR-Tregs, and microbiota consortia) expand Treg numbers but do not correct Foxp3 instability. In chronic inflammation (right), TNF-α/IL-6 and succinate drive ER stress and ubiquitin-mediated FOXP3 degradation, leading to exFOXP3 Th17 conversion and disease progression (IBD, CRC, celiac disease). Predictive biomarkers (succinate, TSDR methylation, FOXP3Δ2/FL ratio) stratify patients, while next-generation strategies—gut-restricted HDAC inhibitors and exhaustion-resistant CAR-Tregs—aim to actively stabilize FOXP3 rather than merely increase Treg counts.

Addressing these three questions—stability, engineering, and stratification—will not only accelerate the bench-to-bedside journey of Tregs therapies but also establish a new paradigm for precision immunotherapy in chronic inflammatory and neoplastic diseases of the gut. The key future directions and proposed framework are illustrated in Figure 6. The core stability-adaptation paradox and its therapeutic implications are synthesized in Figure 7.

### Box 1 The Stability-Adaptation Paradox: A Unifying Framework for Intestinal Treg Biology

The intestinal environment imposes a fundamental trade-off on regulatory T cells (Tregs). Their ability to adapt to local cues—microbial metabolites, dietary retinoic acid, and tissue damage signals—enables specialized functions such as RORγt⁺ and GATA3⁺ differentiation and amphiregulin-mediated mucosal repair. However, the same plasticity becomes a liability under chronic inflammation, where metabolic and cytokine signals destabilize FOXP3, driving pathogenic exFOXP3 Th17 conversion. This stability-adaptation paradox explains why increasing Treg numbers alone often fails therapeutically and points toward a new paradigm: preserving FOXP3 protein integrity, not merely expanding Treg counts, is the key to restoring intestinal immune tolerance as summarized in Table 4.

Current Tregs-based therapies (low-dose IL-2, adoptive polyclonal Tregs, CAR-Tregs) primarily expand Treg numbers but do not correct FOXP3 instability. Consequently, expanded Tregs can convert into pathogenic effectors in the inflammatory milieu. Next-generation strategies must shift from quantitative expansion to qualitative stabilization—e.g., blocking succinate-induced FOXP3 degradation (OGDHc activation), enhancing Hrd1/TMED4 cytoprotective pathways, or engineering CAR-Tregs with enforced FOXP3 co-expression.

Although the intestine—given its substantial microbial and metabolic exposure—offers the clearest illustration of this paradox, the underlying principle is broadly applicable to tissue-resident Treg populations across various organs.

### Testable Hypotheses for Future Validation

### Hypothesis 1 (Metabolite-driven instability)

In IBD patients, colonic mucosal succinate concentration correlates inversely with FOXP3 protein level (but not FOXP3 mRNA) and positively with the frequency of exFOXP3 Th17 cells.

Test: Prospective cohort study measuring succinate by targeted metabolomics and FOXP3/exFOXP3 by flow cytometry/IHC in paired biopsies.

### Hypothesis 2 (Stability-enhancing intervention)

Pharmacological stabilization of FOXP3 (e.g., by inhibiting succinate-induced degradation via OGDHc activation or by enhancing TMED4 function) is more effective than IL-2-based expansion in suppressing chronic colitis and preventing exFOXP3 conversion.

Test: Head-to-head comparison in Cd4CreFOXP3 reporter mice with adoptive transfer of Tregs pre-treated with stabilizer vs. IL-2.

### Hypothesis 3 (Engineered Tregs resilience)

CAR-Tregs co-expressing FOXP3 (as in Henschel et al., 2023) maintain lineage stability, suppressive function, and exhaustion resistance in the inflamed intestinal niche for longer than conventional CAR-Tregs, without malignant transformation.

Test: Long-term (> 6 months) safety and efficacy study in humanized mouse models of IBD, tracking FOXP3 retention, PD-1/TOX expression, and clonal expansion.

## 9. Conclusion

Tregs play a non-redundant role in intestinal immune homeostasis, yet their therapeutic potential has been constrained by a fundamental paradox: the same plasticity that enables tissue adaptation and repair in the steady state renders FOXP3 vulnerable to degradation in the inflamed gut. In this review, we have summarized the molecular basis of this stability-adaptation dilemma, ranging from stabilizing cues such as short-chain fatty acids, bile acids, and retinoic acid, to destabilizing signals including succinate, TNF-α, and IL-6 that promote FOXP3 succinylation loss, ubiquitination, and subsequent proteasomal turnover, ultimately driving the generation of pathogenic exFOXP3 Th17 cells. We have also evaluated current Treg-directed modalities—adoptive transfer, low-dose IL-2 and its engineered variants, CAR-Tregs, and microbial consortia and suggest that their limited and variable efficacy can be traced to a common deficiency: they augment Treg numbers without effectively preserving FOXP3 protein integrity in the inflammatory milieu.

On this basis, we advocate a conceptual shift from quantitative expansion to qualitative stabilization. Future strategies must prioritize FOXP3 stabilization, whether by blocking succinate-induced degradation, enhancing Hrd1/TMED4 cytoprotective pathways, engineering exhaustion-resistant CAR-Tregs with enforced FOXP3 co-expression, and developing biomarkers (succinate, TSDR methylation, FOXP3Δ2/FL ratio) for patient stratification. Addressing these challenges will transform Tregs-based immunotherapy from a proof-of-concept into a precision medicine for inflammatory bowel disease, colorectal cancer, and related intestinal disorders.

## Figures and Tables

**Table 1:** Tregs Subsets: Phenotypic and Functional Heterogeneity

Subset	Origin	Key Markers	Stability	Primary Function	Induction/Maintenance Factors	Alterations in Intestinal Diseases	References
**tTregs (nTregs)**	Thymus	CD25⁺, FOXP3⁺, Helios⁺, Nrp1⁺	High (epigenetically stable)	Self-antigen tolerance; systemic immune homeostasis	Thymic selection; IL-2	overall Treg dysfunction in IBD (instability, impaired function)	15, 21-23, 44, 46, 96
**pTregs**	Peripheral (naïve CD4⁺ T cells)	FOXP3⁺, Helios⁻, Nrp1⁻	Moderate (dependent on TGF-β)	Tolerance to environmental antigens (microbiota, diet)	TGF-β, IL-2, retinoic acid	Induction impaired by dysbiosis; reduced in active IBD	16-18, 41, 42, 83, 84
**iTregs**	*In vitro* induction	FOXP3⁺, Helios⁻, Nrp1⁻	Low (incomplete CpG demethylation)	Research tool; functional relevance controversial	TCR stimulation + TGF-β, IL-2	unstable in inflammatory environment	19, 20, 96
**Tr1**	Peripheral	FOXP3⁻, IL-10⁺, TGF-β⁺	N/A	IL-10/TGF-β-mediated suppression; compensates for FOXP3⁺ Treg deficiency	IL-27, IL-10	Compensatory expansion in colitis, but impaired by ER stress	24, 25, 55, 97, 119
**RORγt⁺ Tregs**	Intestine (pTregs)	FOXP3⁺, RORγt⁺, Helios⁻, Nrp1⁻	Moderate	Suppresses Th1/Th17 inflammation; stabilizes FOXP3	Microbiota colonization; RORγt⁺ APCs	Reduced proportion in active IBD	30, 31, 83, 84, 87
**GATA3⁺ Tregs**	Intestine (mostly nTregs)	FOXP3⁺, GATA3⁺, ST2⁺	High	Responds to tissue damage signals (IL-33); promotes Treg stability	IL-33, TCR signal	Expanded by IL-33 during intestinal inflammation; may be increased in IBD	32, 71, 84, 88

**Table 2:** Tregs-targeted immunotherapeutic strategies for colorectal cancer

Strategy Category	Target/ Molecule	Example Agent	Mechanism	Development Stage	Key Challenge	Refs
**Depletion**	CD25	Daclizumab, Basiliximab	Deplete CD25ʰⁱ Tregs	Preclinical / Early clinical (discontinued in oncology)	Also depletes activated Teff	103, 104, 156
	CCR4	Mogamulizumab	Deplete skin/gut-homing Tregs	Approved in CTCL; under investigation in CRC	Limited to CCR4⁺ subset	157-159
	GITR	DTA-1 (agonist)	Deplete Tregs + activate Teff	Preclinical	Agonism vs antagonism unclear	160
	TIGIT	Tiragolumab (Fc-competent)	FcγR-mediated Tregs depletion	Phase II/III	Requires Fc optimization	161
**Functional modulation**	FOXP3 stability	TMED4 inhibitor	Destabilize FOXP3, convert Tregs	Preclinical	Off-target effects	45
	Adenosine pathway	Anti-CD73, A2AR antagonist	Block Tregs-mediated metabolic suppression	Phase I/II	Tumor specificity	162
	PI3Kδ	Idelalisib	Inhibit Tregs proliferation	Preclinical	Sparing Teff	163
	HDAC	Entinostat	Epigenetic modulation of Tregs function	Phase II	Dual effects	164
Combination	PD-1 + Treg depletion	Anti-PD-1 + low-dose anti-CD25	Synergistic anti-tumor immunity	Preclinical	Toxicity profile	163 164

**Table 3:** Tregs-Targeted Therapeutic Strategies: A Comparative Overview

Strategy Category	Representative Approach	Mechanism	Development Stage	Advantages	Challenges	Refs
Adoptive Tregs Transfer	Autologous/allogeneic polyclonal Tregs	Systemic restoration of immune tolerance	Phase I/II (GvHD, T1D, IBD)	Safety established; infused cells can persist long-term	Variable efficacy; existence of an optimal dose window	114, 115, 117, 118
Optimized Cell Manufacturing	Hypoxia culture; HSF1 stabilization	Enhanced expansion efficiency and stability of Tregs	Preclinical / Process optimization	Higher yield; more stable Tregs	Requires validation of clinical translational value	120, 121
Low-Dose IL-2	IL-2; engineered IL-2 mutants (e.g., IgG-(IL-2N88D) ₂, Fc-IL-2 fusions)	Selective expansion of Tregs	Phase I/II (UC)	Systemic intervention; can significantly expand Tregs	Heterogeneous efficacy; Tregs expansion without clinical improvement in some patients	40, 123, 124
TCR-Tregs	Antigen-specific TCR transduction	MHC-restricted targeting	Preclinical	Signaling closer to physiological state; good stability	MHC restriction; requires screening for functional TCRs	125-129
CAR-Tregs	CARs (e.g., CEA, FliC, IL-23R)	MHC-independent targeting	Preclinical	Flexible target selection; enhanced homing capacity	Tonic signaling and exhaustion risk; stability affected by signaling domain; co-expression of FOXP3 can enhance stability	101, 133, 136-141, 147, 150-153
Fecal Microbiota Transplantation (FMT)	Multi-donor, high-intensity protocol	Restoration of gut ecology and metabolites	Phase II (UC)	Multi-target, holistic regulation	Donor-dependent; complex mechanism	143, 144
Microbial Consortium	GUT-108 (17 strains)	Synergistic induction of IL-10⁺ Tregs	Preclinical (colitis model)	Rationally designed; can reverse established colitis	Safety and stability in humans need validation	145
Microbiota Editing	Bacteriophage-delivered base editors	In-situ modification of Tregs-inducing genes	Proof-of-concept (animal)	Precise; programmable	Safety and delivery efficiency need validation	146

**Table 4:** FOXP3 stability switch: steady state versus chronic inflammation

Condition	Steady state	Chronic inflammation (IBD, CRC)
**Key Signals**	SCFAs, isoalloLCA, retinoic acid; low TNF/IL-6	Succinate ↑, TNF-α, IL-6; ER stress
**FOXP3 Stability**	**Stable** (epigenetic locking; Hrd1/TMED4 protective)	Unstable (succinylation loss → ubiquitination → degradation)
**Tregs Functional Output**	Immunosuppression (IL-10, CTLA-4) + Tissue repair (AREG)	exFOXP3 Th17 conversion (IL-17A, IFN-γ) + Loss of suppression
**Outcome**	Tolerance, barrier integrity	Colitis, tumor immune escape

## Data Availability

All data supporting the summary described in the manuscript are available in the article and from the corresponding authors upon reasonable request. Source data are provided with this paper.

## Abbreviations

A2AR: A2A adenosine receptor
APC: antigen-presenting cell
AREG: amphiregulin
ARG2: arginase 2
BLIMP1: B lymphocyte-induced maturation protein 1
cAMP: cyclic adenosine monophosphate
CAR: chimeric antigen receptor
CD: cluster of differentiation
CNS: conserved non-coding sequence
CpG: 5'-C-phosphate-G-3'
CRC: colorectal cancer
CRISPR: clustered regularly interspaced short palindromic repeats
CTLA-4: cytotoxic T lymphocyte-associated antigen 4
DC: dendritic cell
DLST: dihydrolipoamide S-succinyltransferase
Eomes: eomesodermin
ER: endoplasmic reticulum
FcγR: Fc gamma receptor
FL: full-length
FMT: fecal microbiota transplantation
FOXP3: forkhead box protein 3
GATA3: GATA binding protein 3
GITR: glucocorticoid-induced TNFR-related protein
GLUT1: glucose transporter 1
GPR109A: G protein-coupled receptor 109A
GvHD: graft-versus-host disease
HDAC: histone deacetylase
HLA: human leukocyte antigen
Hrd1: HMG-CoA reductase degradation protein 1
HSF1: heat shock factor 1
IBD: inflammatory bowel disease
iCasp9: inducible caspase 9
IDO: indoleamine 2,3-dioxygenase
IFN γ: interferon γ
IL: interleukin
IRE1α: inositol-requiring enzyme 1α
LAG-3: lymphocyte activation gene 3
LPS: lipopolysaccharide
MGCP: mannan/β-glucan
MHC: major histocompatibility complex
mLN: mesenteric lymph node
mTOR: mammalian target of rapamycin
MyD88: myeloid differentiation primary response 88
NF κB: nuclear factor κB
NRF2: nuclear factor erythroid 2 related factor 2
Nrp1: neuropilin 1
OGDHc: 2 oxoglutarate dehydrogenase complex
PD 1: programmed cell death protein 1
PI3K: phosphoinositide 3 kinase
PKA: protein kinase A
PSA: capsular polysaccharide of Bacteroides fragilis
RALDH2: retinaldehyde dehydrogenase 2
RORγt: RAR related orphan receptor γt (also RORC2)
RQR8: combination of CD20 and CD34 epitopes (safety switch)
scFv: single chain variable fragment
SCFA: short chain fatty acid
STAT5: signal transducer and activator of transcription 5
ST2: IL 33 receptor (IL1RL1)
T bet: T box transcription factor TBX21
TCR: T cell receptor
Teff: effector T cell
TGR5: Takeda G protein coupled receptor 5
Th: T helper cell
TIGIT: T cell immunoreceptor with Ig and ITIM domains
TIM3: T cell immunoglobulin and mucin domain containing protein 3
TI Treg: tumor infiltrating regulatory T cell
TLR: Toll like receptor
TMED4: transmembrane p24 trafficking protein 4
TME: tumor microenvironment
TNF α: tumor necrosis factor α
TOX: thymocyte selection associated high mobility group box protein
Tr1: type 1 regulatory T cell
Treg: regulatory T cell
TSDR: Treg specific demethylated region
tTreg: thymus derived regulatory T cell (also nTreg)
UC: ulcerative colitis
